# Optimizing genomic diversity assessments for conservation of *Bromus auleticus* (Trinius ex Nees) using individual and pooled sequencing

**DOI:** 10.1371/journal.pone.0325548

**Published:** 2025-06-25

**Authors:** Luciana Gillman, Federico Condón, Cesar Petroli, Mercedes Rivas

**Affiliations:** 1 Departamento de Sistemas Agrarios y Paisajes Culturales, Centro Universitario Regional del Este, Universidad de la República, Treinta y tres y Rocha, Uruguay; 2 INIA Estanzuela, Unidad de Semillas y Recursos Fitogenéticos, Instituto Nacional de Investigación Agropecuaria (INIA), Colonia, Uruguay; 3 Centro Internacional de Mejoramiento de Maíz y Trigo (CIMMYT), El Batán, Texcoco, México; 4 Departamento de Biología Vegetal, Facultad de Agronomía, Universidad de la República, Montevideo, Uruguay; KGUT: Graduate University of Advanced Technology, IRANISLAMIC REPUBLIC OF

## Abstract

*Bromus auleticus*, a valuable forage grass native to the Pampa biome, is currently undergoing genetic erosion. Therefore, it is essential to assess appropriate methodologies for developing population genomic studies that will contribute to the conservation of this genetic resource. In this study, we evaluated five accessions using two genotyping strategies: individual sequencing (ind-seq) and pooled sequencing (pool-seq). To assess methodologies effectiveness, the correlation between allele frequencies calculated using each approach was investigated, as well as genetic diversity and population structure. These comparisons explicitly accounted for the potential effects of factors such as sample size, missing data, sequencing depth, and minor allele frequencies. The highest values of frequencies concordance and percentage of SNPs in common between ind-seq and pool-seq were achieved using a sample size of 30–60 plants per accession. These values were obtained with a maximum missing data threshold of 10% and a less strict minimum allele frequency threshold for pool-seq (0.01) compared to ind-seq (0.05). Pool-seq required a higher sequencing depth per accession (4.8 million reads) compared to ind-seq (0.9 million reads) to achieve similar allele frequencies. Pools of 50 individuals yielded the highest number of polymorphic sites, averaging over 9,000 per accession at a sequencing depth of 4.8 Mr. Under these conditions, pool-seq consistently resulted in an average of 0.09 higher expected heterozygosity and a 0.24 lower allelic richness compared to ind-seq in all accessions. Population structure inferred with both methodologies confirmed the outcrossing nature of *B. auleticus* and aligned with the geographical origin of each accession. The average inbreeding coefficient of 0.2 evidence inbreeding, which highlights the importance of conservation efforts for this valuable plant genetic resource. Based on these findings, we propose two workflows for conducting population genomics studies on *Bromus auleticus*.

## 1. Introduction

*Bromus auleticus* is a perennial grass species endemic to the Pampa biome, valued for its high nutritional content and potential for winter forage production [1–3]. This outcrossing species has a hexaploid genome (2n = 6x = 42) and an estimated genome size of approximately 18 Gb [4,5]. Previous studies have revealed substantial intra- and inter-population genetic diversity, and a phenotypic variation associated to eco-geographical conditions that define distinct ecotypes. These studies have employed an assortment of approaches, including morpho-phenological descriptors [6,7], as well as molecular markers such as isoenzymes [8–10] and Random Amplified Polymorphic DNA (RAPD) [10,11]. Despite its ecological and agronomic importance, *Bromus auleticus* is now rare in native grasslands and nearly extinct in agricultural landscapes [1]. To support its conservation and use, the Germplasm Bank of the National Institute of Agricultural Research (INIA) in Uruguay conserves 82 characterized accessions of *B. auleticus*, which have been phenotypically evaluated [6,12].

Domestication efforts for *Bromus auleticus* have primarily focused on the evaluation and selection of wild populations, alongside the development of technologies for seed sowing, cultivation, and harvesting. Additionally, a limited number of cultivars of this species have been derived from the selection of highly productive populations and are currently utilized on a small scale [13–15]. Despite the progress achieved, key challenges remain, particularly related to seed production and the enhancement of seedling vigor during the early developmental stages of *B. auleticus*.

Advancements in next-generation sequencing (NGS) technologies have revolutionized the study of genetic diversity in non-model species, such as *B. auleticus*. These technologies do not require prior sequence information and enable the generation of high-throughput genotypic data [16]. DArTseq, a widely used genotyping approach, combines DNA restriction enzyme digestion with NGS to generate informative genomic fragments. The enzymes employed, along with other experimental conditions, are optimized for each species to preferentially target active genes while minimizing the representation of repetitive elements [17–19]. Effective implementation of this methodology requires careful consideration of several factors, including the study objectives, species biology, and the availability of economic, human, and computational resources. Key aspects of experimental design include the selection of the sampling strategy [20–24], determination of the optimal number of individuals and populations [20,25–27], sequencing depth [21,28], filtering parameters of polymorphic sites (e.g., minor allele frequency and missing data thresholds) [29–32], and the choice of the appropriate analytical software [21,33,34]. To optimize the study design to specific species and research context, preliminary studies are recommended [25,29] to improve the reliability and interpretability of the resulting data.

In population genetics, the number of populations sampled is contingent upon the genetic structure of the species. Since unique alleles may occur in different populations, a broad sampling strategy is required to capture as much genetic diversity as possible [35,36]. Within populations, it is important to balance sampling effort: too few individuals may result in inaccurate estimates, while excessive sampling can be resource-intensive [25,37]. Optimal sample size depends on multiple biological factors, including species’ evolutionary history, mating system, degree of geographic isolation, and the occurrence of bottleneck events [25,36,38]. In the context of germplasm bank collections, accurate assessments of genetic diversity typically require many molecular markers though multiple individuals across most preserved populations.

The pool-seq approach has been used in studies involving several populations. This method comprises the consolidation of DNA from multiple individuals into a composite sample, reducing costs and labor while maintaining the potential for high-throughput analysis [20,39–41]. The efficiency of pool-seq is contingent upon several factors such as sequencing depth, the number of individuals per pool and the relative DNA representation of those individuals within the pool [42]. Pool-seq has demonstrated its efficacy in the estimation of allele frequencies, population structure, and genetic diversity when showing high concordance with individual sequencing (ind-seq) results. Most studies have demonstrated a favorable cost-benefit ratio [20,21,23,34,28,43]. Moreover, the advantages of pool-seq were established concerning microsatellites, with promising results for assessing genetic diversity and landscape genetics [27,44,45]. However, the employment of pool-seq is not recommended for studies requiring individual-level data, such as parentage analysis [42].

This research may provide an opportunity to refine sampling strategies for the *Bromus* genus, an area that has been relatively underexplored in genomic studies [46–49]. Standardizing sampling and sequencing procedure for *B. auleticus* could enhance the effectiveness of both conservation and breeding programs [50]. To our knowledge, comparison between ind-seq and pool-seq in polyploid species have been limited to a single study in the autotetraploid *Arabidopsis kamchatica subsp. kamchatica*, using only eight genes [22], highlighting a gap that this research aims to address. The primary objective of this study is to compare the use of both methods, ind-seq and pool-seq, to analyze the genetic diversity of *Bromus auleticus*. We assess the effects of sample size, missing data cut-offs, sequencing depth, and minor allele frequency threshold on allele frequency concordance, genetic diversity estimates, and population structure. Specifically, we evaluate how sample size influences ind-seq results and the impact of both sample size and sequencing depth on pool-seq outcomes. Concordance in allele frequency estimates between methods is quantified using the concordance correlation coefficient, and the proportion of shared SNPs. Genetic diversity and population structure are analyzed using expected heterozygosity, allelic richness, Nei’s distance, molecular variance analysis, and multidimensional scaling. We hypothesized that, with adequate sample size, sufficient sequencing depth, and replicates, pool-seq could provide genetic estimates comparable to those obtained with ind-seq, offering a cost-effective alternative for large-scale genetic studies in *B. auleticus.*

## 2. Methodology

### 2.1 Sampling

**Fig 1:**
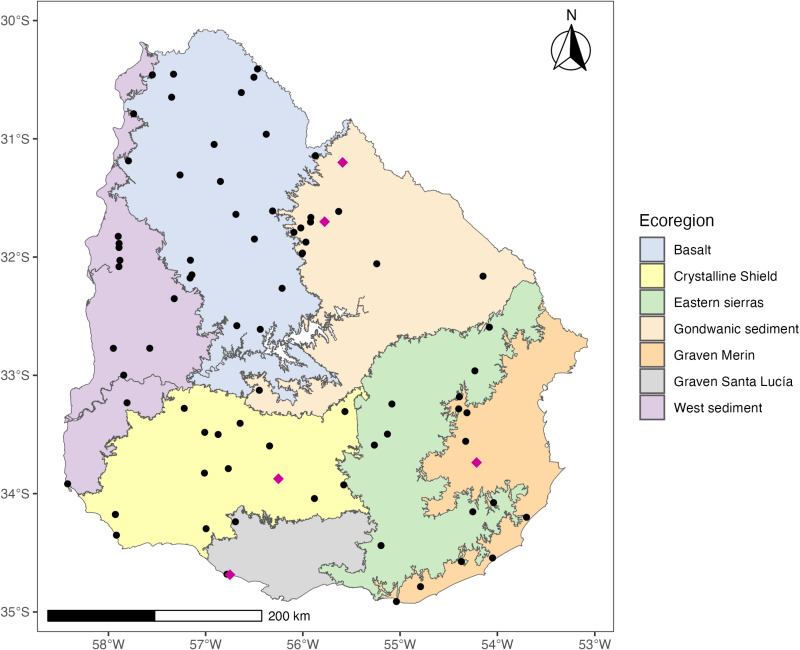
Geographical map of Uruguay showing the spatial distribution of the 82 accessions conserved in the INIA germplasm bank. The purple diamonds indicate the five accessions evaluated in this study. Black circles represent the 77 accessions pending genomic evaluation. The map includes ecoregions according to the classification framework established by Brazeiro et al. (2012) [51]. The colour scheme is delineated in the accompanying legend. The graph was generated using the “ggplot2” package in R [53].

Five accessions of *Bromus auleticus* were selected from the germplasm bank of INIA (National Institute of Agricultural Research) in Uruguay. The selection of these accessions was based on the presence of contrasting phenotypes and their respective ecoregions of origin within the country [6,51]. These accessions were originally collected by researchers from INIA between 1970 and 2009, as previously described by Condón et al. (2017) [6] (Fig 1; S1 Table). This collection was carried out prior to Uruguay’s ratification of the Nagoya Protocol on June 24, 2014, under Law Nº 19.227, which came into effect on October 12, 2014; therefore, no permits were required.

**Fig 2:**
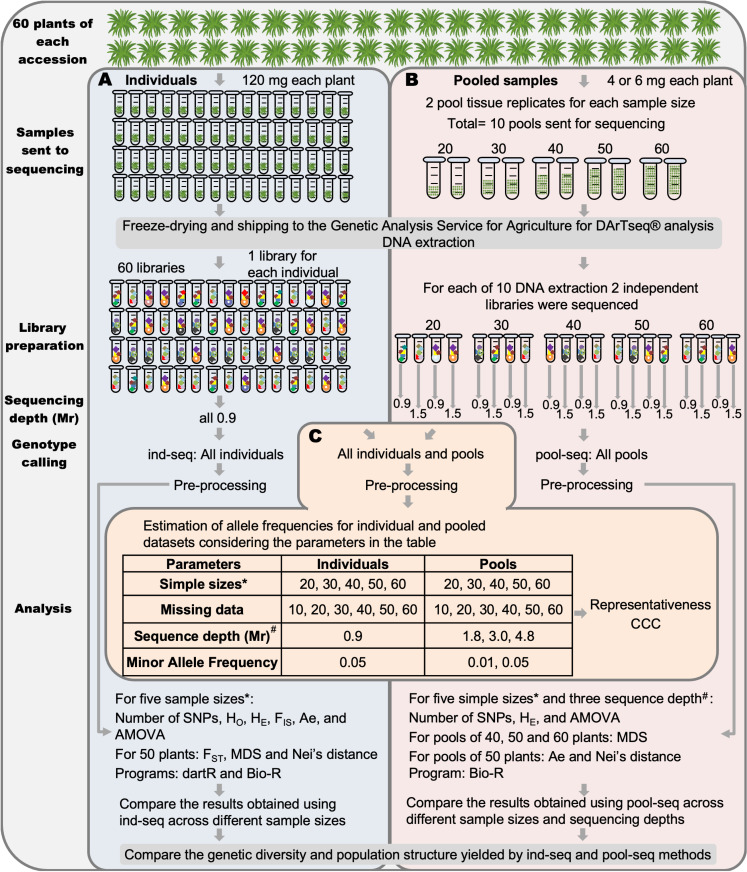
Diagram illustrating the procedures and parameters analyzed, using one accession as an example. (A.) Individual sequencing (ind-seq) (blue background) involved individual sample collection, unique barcoding, sequencing at 0.9 Mr depth, individual SNP calling, and analysis with dartR and Bio-R. (B.) Pooled sequencing (pool-seq) (pink background) involved two tissue replicates, two libraries per replicate, sequencing at two depths, pooled SNP calling, and analysis with Bio-R. (C.) Common SNP calling (orange background) is employed to compare allele frequencies between paired individuals and pools with the same sample size and missing data. The minimum allele frequency and sequencing depth used in the individual SNP dataset remained constant. They were compared with pooled datasets based on two MAF and three sequencing depths. See the step-by-step protocol for more details [54]. This diagram was created using Microsoft PowerPoint.

From each accession, 60 seedlings were randomly selected (Fig 2A). Seed germination was conducted according to the standards established by the International Seed Testing Association [52]. The seedlings were then transplanted into trays, and once sufficient leaf tissue had developed, both individual and pooled samples were collected. A 120 mg fresh leaf sample was obtained from each of the 60 plants of the five accessions, as per the service provider’s specifications. Initially, leaf tissue from 20 randomly selected seedlings was pooled to create the 20-sample pool. An additional 10 seedlings were included to generate the 30-sample pool, and this process was repeated to produce the 40-, 50-, and 60-sample pools. Each pool contained 4 mg of leaf tissue per plant, except for the 20-sample pool, which included 6 mg of tissue per plant. Pool compositions were recorded for comparison with individual samples data. To minimize biases originating from disparities in DNA representation among individuals in the pool, duplicate pools were constructed following established protocols (Fig 2B) [23,42]. To ensure samples integrity, all tubes were kept cold during pool preparation. Leaf tissue samples were lyophilized for 72 hours in a NovaDryer-F104 Senova lyophilizer.

### 2.2 Genotypic data

Lyophilized samples (120 mg of leaf tissue) were sent to the high-throughput genotyping platform SAGA (Servicio de Análisis Genético para la Agricultura, SAGA), where DNA was extracted using the modified cetyltrimethylammonium bromide (CTAB) method [55,56]. Library preparation followed the DArTseq® protocol [18,57], digesting DNA with the restriction enzymes *PstI* and *MseI* [17,57]. Adapters were ligated to the fragments generated previously. The adapters sequence included barcodes for sample identification, Illumina ﬂowcell attachment, and the polymerase chain reaction (PCR) primer sequences. PCR amplification was performed on each sample, and the products were pooled into an equimolar mixture. Sequencing was performed on an Illumina Novaseq 6000 platform, generating sequences up to 83 bases in length. Each individual sample was sequenced to an approximate depth of 0.9 million reads (Mr). For each sampled pool, two libraries were prepared: the first was sequenced at a depth equivalent to that of the individual samples, while the second was sequenced at a depth of 1.5 Mr per pool (Fig 2B). The quality filtering and SNP calling procedure was performed in the DArTsoft14 software developed by DArT P/L [18]. Quality filtering was based on Phred quality scores and marker reproducibility. A total of 296 samples fulfilled the specified quality filtration standards. Among them, 58 individual samples were from accessions 50 and 87, 60 individual samples were from the three remaining accessions, and 100 were pooled samples (20 for each accession). Single nucleotide polymorphisms (SNPs) were discovered through a de novo calling approach based on fragment sequences from the genotyped samples [18]. To achieve the objectives of this study, three SNPs calling processes were implemented. The first included all samples (Fig 2C) and generated two types of markers report: one with binary presence-absence or score data (S2 Table), and another with allele counts per sample (S3 Table). These reports encompassed 130,261 markers with an initial missing data (MD) of 29%. The second calling process utilized 196 individual sequence (Fig 2A) and produced a score report with 156,801 markers with a 27% MD rate in a score report (S4 Table). The final colling, based on the 100 pooled samples (Fig 2B), yielded a count marker report with 184,373 markers and 43% of MD rate (S5 Table).

### 2.3 Comparison of polymorphic sites and allele frequencies obtained with individual and pooled samples

#### 2.3.1 Allele frequency calculation from individual samples.

The same plants were used for the comparison of individuals and pooled sequencing datasets for each sample size. Based on the data in S2 Table, allele frequencies were calculated for each accession (Fig 2C). Allele frequencies (p and q) were calculated in the R program [58] using the following formula:


$$p=\frac{AA+\frac{1}{2}Aa}{aa+Aa+AA}$$

q=1−p

Where AA is the number of homozygous individuals for the major allele, Aa is the number of heterozygous individuals, and aa is the number of homozygous individuals for the minor allele. SNPs with a minor allele frequency (MAF) of 0.05 or lower were discarded. The variability of both the number of SNPs detected and the allele frequencies within each accession was analyzed, considering different sample sizes and maximum missing data thresholds (MD < 10, 20, 30, 40, 50, 60%). The command lines used are available in a repository [59].

#### 2.3.2 Allele frequencies estimation from pooled samples.

For comparison depth sequencing purposes, data from the two tissue replicates (sequenced at 0.9 or 1.5 Mr) were merged to calculate allele frequencies at sequencing depths of 1.8 and 3.0 Mr, using the count data provided in S3 Table. Consoliding all replicates of each accession resulted in a third sequencing depth of 4.8 Mr (Fig 2B). These calculations were performed using the count file and the corresponding R command lines [59].

Allele frequencies for each sequencing depth were calculated as follows:


$$p=\frac{n\_A}{n\_A+n\_a}$$

q=1−p

Where n_A is the number of times the major allele was counted in the accession and n_a is the number of times the minor allele was recorded within the same accession. The effects of an incremental increase in both the maximum missing data threshold (from 10% to 60%) and the sample size (from 20 to 60) were evaluated. Given previous studies demonstrating the positive influence of increasing sequencing depth on the precision of allele frequency estimates in pool-seq, its effect was analyzed here using three different depths (1.8, 3.0, and 4.8 Mr) [21,42]. Two MAF thresholds were tested: a standard threshold of 0.05 and a less strict threshold of 0.01.

#### 2.3.3 Comparison of SNPs obtained from individual and pooled sample data.

For each accession, a pairwise comparison of the number of shared SNPs and allele frequencies was performed between the individual and pooled datasets (Fig 2C) across the following variables:

- Same sample size and missing data rate.
- MAF ≤ 0.05 in individuals with MAF ≤ 0.01 and 0.05 in pooled datasets
- Sequence depth of 0.9 Mr in individuals with three sequencing depths of pooled datasets (1.8, 3.0, and 4.8 Mr).

To assess whether the number of shared SNPs in the pooled samples reliably estimates the number of SNPs detected in individual samples, we calculated the Representativity (%).


$${\text{Representativity}\;(\%)}_{asmjk}\;=\frac{\;{Shared\;SNPs}_{asmjk}}{{Number\;of\;SNPs}_{{ind}_{asm}}}\times 100$$

$${Shared\;SNPs}_{asmjk}={count(SNPs}_{{ind-seq}_{asm}}⋂{SNPs}_{{pool-seq}_{asmjk}})$$

Since ${Shared\;SNPs}_{asmjk}$ is the number of markers in common between individual and pooled sequencing data for each factor combination, *a* is accession (24, 28, 50, 87, 88), *s* the sample size of individuals and pooled datasets (20, 30, 40, 50 and 60), *m* is the missing data percentage threshold applied to individuals and pooled datasets (10, 20, 30, 40, 50, 60%), *j* the sequencing depth of pool sequencing (1.8, 3.0 and 4.8 Mr) and *k* is the minor allele frequency applied to pooled dataset (0.01, 0.05). ${Number\;of\;SNPs}_{{ind}_{asm}}$ is the number of SNPs in individual sequencing data for each factor combination. While ${SNPs}_{{ind-seq}_{asm}}\;$and ${SNPs}_{{pool-seq}_{asmjk}}$ are the molecular marker id detected in individuals and pooled sequencing data for each factor combination, respectively.

Also, the Concordance Correlation Coefficient (CCC) was calculated to assess the relationship between frequencies estimated from individual and pooled sequencing data. This statistical measure quantifies both the accuracy and precision of the relationship, indicating how well the data points align with perfect concordance, defined as the 45° line through the origin. It has been used in other studies for measure methods comparison [21,60–62]. The CCC was calculated using the “epi.ccc” function from the “epiR” package in R [60,63].


$${CCC}_{asmjk}\;=\frac{2\;\sigma_{{{ind}_{asm}\;pool}_{asmjk}}}{\sigma_{{ind}_{asm}}^{2}+\sigma_{{pool}_{asmjk}}^{2}+{\;(\mu_{{ind}_{asm}}-\mu_{{pool}_{asmjk}})}^{2}\;}$$

Where $\sigma_{{{ind}_{asm}\;pool}_{asmjk}}$ is the covariance, $\sigma_{{ind}_{asm}}^{2}$ and $\sigma_{{pool}_{asmjk}}^{2}$ are variances, and $\mu_{{ind}_{asm}}\;$ and $\mu_{{pool}_{asmjk}}$ are means of allele frequencies calculated from individual and pooled sequencing data, respectively. Since *a*, *s*, *m*, *j*, and *k* are the same variables explained for Representativity (%).

### 2.4 Diversity and population structure analysis using individual sequencing dataset (ind-seq)

To read the data in S4 Table, we used the “gl.read.dart” function from the “dartR” package in R [64]. To maintain the traceability referenced in Section 2.1, plant genotypes were assigned using the “gl.keep.ind” function from the same package. The loci with MD < 10% were retained using the “gl.filter.callrate” function of “dartR” package, this threshold was selected based on the highest Representativity and CCC obtained, see section 3.1 Results. Subsequently, a MAF threshold of 0.05 was then applied using the “gl.filter.maf” function from the “dartR” package.

To analyze and contrast the intra-accession diversity for each sample size, the number of polymorphic sites (number of SNPs), the average observed heterozygosity (H_O_), the average expected heterozygosity (H_E_), and the average inbreeding coefficient (F_IS_) were calculated using the “gl.report.heterozygosity” function of “dartR”. The “genlight” file was converted to “genind” using the “gl2gi” function from the same package, and the average allelic richness (Ae) per accession was calculated using the “allel.rich” function from the R package “PopGenReport” [65] (Fig 2A). The command lines used to calculate these parameters are available in the repository [59].

To obtain and compare the population structure for each sample size, the molecular variance analysis (AMOVA) was conducted using BIO-R Version 3.2 [66]. To calculate the pairwise genetic distances between accessions, the “gl.fst.pop” and “gl.dist.pop” functions from the R package “dartR” were utilized. The first function calculates the inbreeding coefficient (F_ST_), and the second perform Nei analysis. Additionally, multidimensional scaling (MDS) was obtained in BIO-R using Roger’s distance (Fig 2B). The explanatory variances of each MDS component were calculated from the eigenvalues obtained with the “cmdscale” function from the “stats” package in r. For these analyses, we used the dataset of 50 individuals according to the results in Sections 3.1, 3.3.1, 3.4.1.

### 2.5 Diversity and population structure analysis with pooled sequencing dataset (pool-seq)

The data presented in S5 Table was filtered using MD and MAF thresholds of 10% and 0.01, respectively. This filtration process was based on the Representativity and CCC results presented in Section 3.1. To obtain the number of SNPs, H_E_, and AMOVA analyses were conducted using BIO-R Version 3.2 [66] across all sample sizes and sequencing depths. To assess population diversity and structure of *B. auleticus* and enable comparisons with individual-level data, the number of effective alleles (Ae) and Nei’s genetic distance were calculated with the same software using data from pools of 50 individuals sequenced at 4.8 Mr. Additionally, multidimensional scaling (MDS) analysis, based on Roger’s distance, was conducted using data from the 40, 50 and 60 sample size pools sequenced at 4.8 Mr (Fig 2B). The explanatory variances of the MDS were calculated as described in Section 2.4.

### 2.6 Comparison of diversity and population structure obtained with ind-seq and pool-seq

Delta H_E_ (ΔH_E_) was calculated for each sample size and pool sequence depth.


$${{\Delta H}_{E\;{pool-seq}_{asj}}\;=H}_{E\;{pool-seq}_{asj}}-\;H_{E\;{ind-se}_{as}}$$

Where $H_{E\;ind-seq}$ is the expected heterozygosity calculated with individual sequencing data, $H_{E\;pool-seq}$ is the expected heterozygosity calculated with pooled sequencing data, *a* is the accession (24, 28, 50, 87, 88), *s* is the sample size of individuals and pooled datasets (20, 30, 40, 50, 50) and *j* is the sequence depth of pooled sequencing (1.8, 3.0, 4.8 Mr). The individual plant sequencing data was obtained from sequencing at 0.9Mr.

A comparison of the Nei’s genetic distance matrices was performed using for individual and pooled sequencing data using the “mantel” function from the “vegan” package in R [67].

### 2.7 Variance analysis and mean comparison

An Analysis of Variance (ANOVA) was conducted to evaluate the effects of the different factors (sample size, missing data, sequencing depth, and minor allele frequency) on average Representativity and CCC for a specific accession, and population diversity statistics. The accessions were treated as repetitions. In all the cases, the data showed homoscedasticity of variance using Levene’s test with the “leveneTest” function from the “car” package in R [68].

The first set of ANOVAs was used to evaluate the effect of sample size, missing data, sequencing depth, minor allele frequency, and a combination of them on Representativity and CCC. The second set of ANOVAs was applied to evaluate the influence of sample size on the number of SNPs, H_O_, H_E_, F_IS_, and Ae in ind-seq data. A third set of ANOVAs assessed the effect of sample size and sequencing depth on the number of SNPs and H_E_ in pool-seq data. Finally, a fourth set of ANOVAs was conducted to assess the effect of sample size and sequencing depth on ΔH_E_.

A general model was applied (one-way ANOVA) [69]:


$$Y_{ij}\;=\;\mu \;+\;\alpha_{i}\;{+\;\in }_{ij}$$

Where $Y_{ij}$ is the *j*-th observation in the *i*-th group, μ is the overall mean of all observations across all groups, $\alpha_{i}$ is the effect of the *i*-th group relative to the overall mean, and $\in_{ij}$ is the random error for the *j*-the observation in the *i*-th group. In all the cases, the null hypothesis (H_0_) was that the groups’ means were equal.

Two-way ANOVA was applied to evaluate the effect of sample size and sequence depth on the number of SNPs detected in pooled sequencing data. The model used was the following:


$$Y_{ijk}\;=\;\mu \;+\;\alpha_{i}\;{+\;\beta_{j}{\;+(\alpha \beta )}_{ij}+\;\in }_{ijk}$$

While $Y_{ijk}\;$ is the *k*-th observation in the *i*-th level of factor A and the *j*-level of factor B, μ is the overall mean, $\alpha_{i}$, $\beta_{j}$, and ${\left(\alpha \beta \right)}_{ij}$ are the effect on the *i*-th level factor A, the *j*-th level factor B and their interaction, respectively, and $\in_{ijk}$ is the random error associated with *k*-th observation. The null hypotheses were that the means are equal across all levels of factors A and B, and that there is no interaction between them.

When ANOVA indicated significant differences, a Tukey’s HSD post-hoc test was conducted using the “TukeyHSD” function from the R “stats” package [58]. A significance level of p < 0.05 was considered for all analyses.

## 3. Results

### 3.1 Comparison of SNPs obtained from individual and pooled sample data

**Fig 3:**
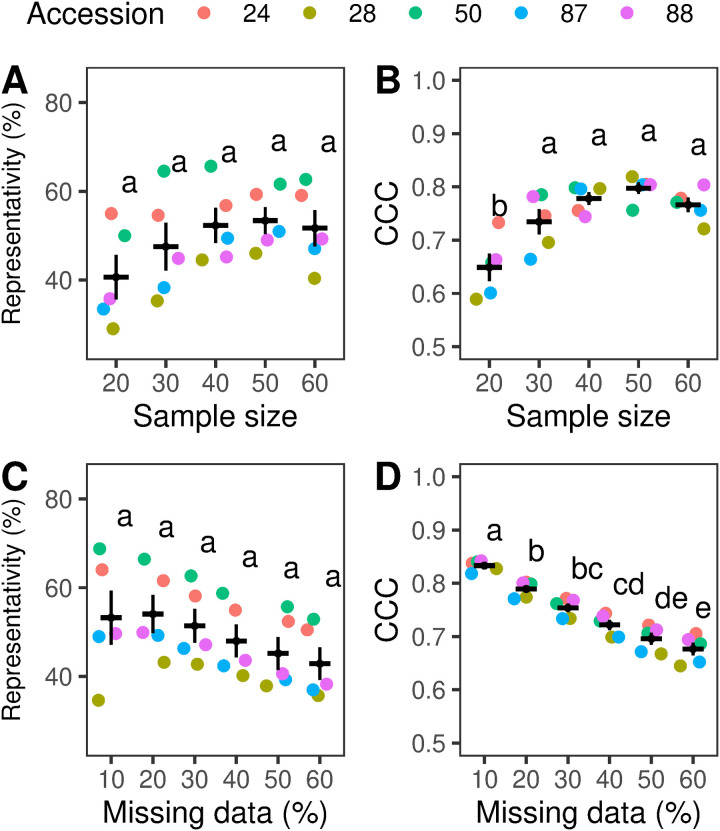
Effect of sample size (A, B) and missing data thresholds (MD) (C, D) on Representativity (%) and Concordance Correlation Coefficient (CCC). Each data point represents the average Representativity or CCC for a single accession, with the color key provided in the legend. For each group, the black horizontal line indicates the means, while the vertical lines represent the standard error. The upper panels (A.) and (B.) illustrate the effect of different sample sizes (20, 30, 40, 50, 60) on Representativity and CCC, respectively. For the same metrics, the lower panels, (C.) and (D.), show the influence of MD thresholds (10, 20, 30, 40, 50, and 60%). Statistical differences between groups, as determined by Tukey’s test (p < 0.05), are indicated by different letters. Plots were generated using the “ggplot2” and “ggpubr” packages in R [53,70].

Both Representativity and CCC, generally increased with larger sample size and decreased with higher levels of MD. The maximum mean values of Representativity and CCC were observed with sample sizes of 30 or more plants, reaching approximately 50% and 0.75, respectively (Fig 3A and 3B). These values were significantly higher than those obtained from 20 individuals (F (4,20) = 10.2, p < 0.001). Regarding MD, the highest mean CCC was achieved with a 10% threshold, approximately 0.85; F (5,24) = 41.5; p < 0.001 (Fig 3C), while the highest mean Representativity values were observed with 10 and 20% (Fig 3D). Detailed results are provided in the S6 Table.

**Fig 4:**
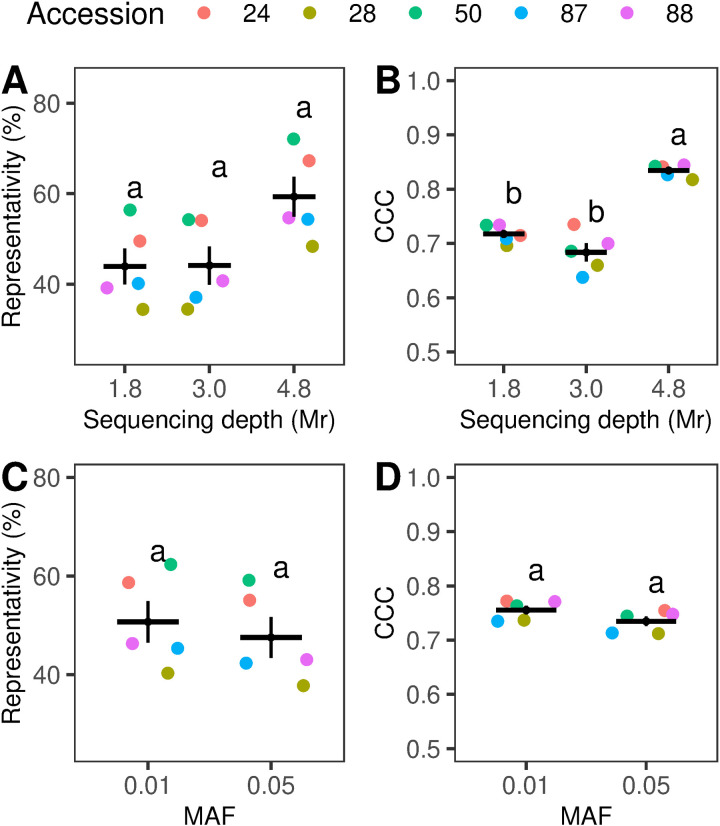
Effect of sequencing depth (Mr) (A, B) and minor allele frequency thresholds (MAF) (C, D) on Representativity (%) and Concordance Correlation Coefficient (CCC). Each dot represents the average Representativity or CCC of an individual accession, with the color coding outlined in the accompanying legend. For each group, the black horizontal line indicates the mean values, while the vertical lines represent the standard deviation. Panels (A.) and (B.) show the effect of sequencing depth (1.8, 3.0, and 4.8 Mr) on Representativity and CCC, respectively. Panels (C.) and (D.) illustrate the effect of MAF thresholds (0.01 and 0.05) on Representativity and CCC, respectively. Statistical differences between groups, as determined by Tukey’s test (p < 0.05), are indicated by different letters. Plots were generated using the “ggplot2” and “ggpubr” packages in R [53,70].

In pooled samples, increasing the sequencing depth and applying a more stringent MAF threshold improved the precision of allele frequency estimations. A sequencing depth of 4.8 million reads resulted in an approximately 18% increase in mean Representativity and a 0.15 increase in CCC (F (2,12) = 51.4, p < 0.001), as illustrated in Fig 4A and 4B. Conversely, increases in MAF led to only minor reductions in mean Representativity and CCC, as shown in Fig 4C and 4D.

**Fig 5:**
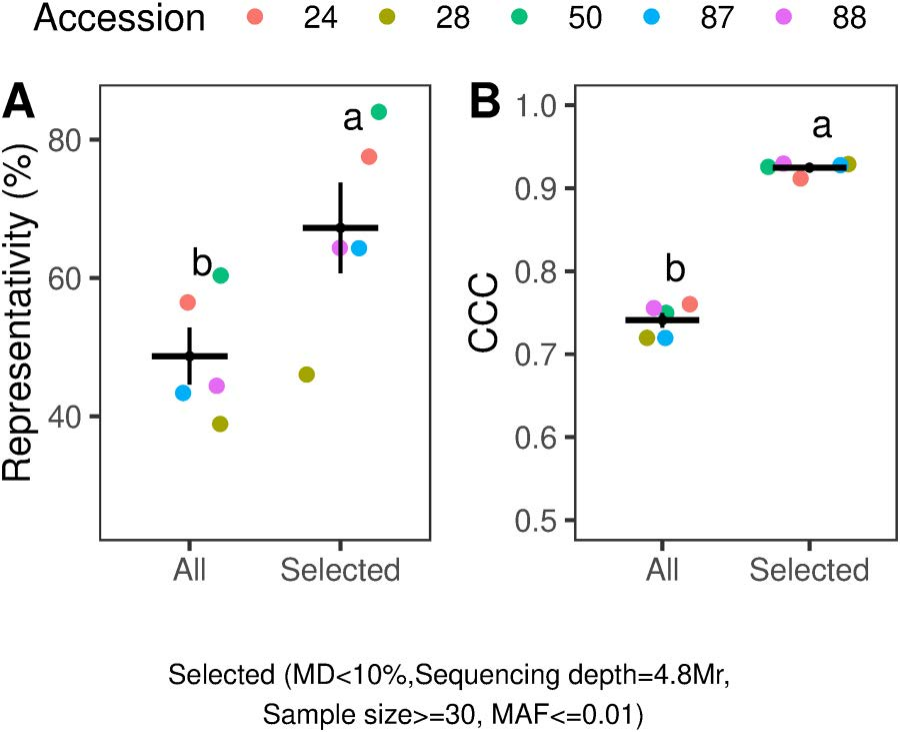
Cumulative effect of optimized factors (Selected) on Representativity (%) and Concordance Correlation Coefficient (CCC). “All” compresses the entire dataset from the S6 Table. The “Selected” dataset includes samples with a minimum size of 30 plants, a missing data (MD) threshold of 10%, a coverage depth of 4.8 Mr for pooled samples, and a MAF threshold of 0.01. Each point represents the average Representativity or CCC of a single accession, with the color coding detailed in the legend. For each group, the black horizontal line indicates the mean value, while the vertical line represents the standard deviation. (A.) Shows the effect of Selected data on Representativity. (B.) Displays the effect of Selected data on CCC. Statistical differences between groups, determined by Tukey’s test (p < 0.05), are indicated by different letters. Plots were generated using the “ggplot2” and “ggpubr” packages in R [53,70].

The optimization of the selected factors resulted in a substantial improvement in both Representativity (approximately 18%, F (1,8) = 5.8, p < 0.05) and CCC (close to 0.2, F (1,8) = 371, p < 0.001) compared to the complete dataset (Fig 5A and 5B).

### 3.2 Diversity and population structure analysis with ind-seq dataset

#### 3.2.1 Effect of sample size on genetic diversity.

**Fig 6:**
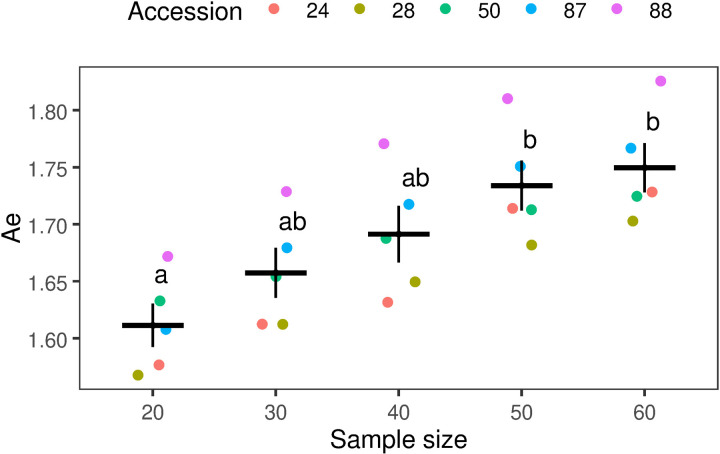
Effect of Sample size on Allele Richness (Ae). Each dot represents a single accession, with the color coding detailed in the legend. For each group, the black horizontal line indicates the mean, while the vertical lines represent the standard deviation. Statistical differences between groups, determined by Tukey’s test (p < 0.05), are indicated by different letters. The plot was generated using the “ggplot2” package in R [53].

The number of SNPs, H_O_, H_E_, and F_IS_ did not show significant sensitivity to variations in sample size. However, the Ae increased with larger sample sizes (Table 1). The average number of SNPs across accessions increased by 15% as the sample size increased from 20 to 60 individuals, although this difference was not statistically significant. In contrast, the mean Ae for sample sizes of 50 or 60 individuals was significantly higher compared to a sample size of 20 individuals, with an increase of more than 0.1 (F (4,20) = 6.6, p < 0.01; Fig 6). Table A in S1 Appendix provides the detailed breakdown of the genetic diversity outcomes for each accession.

**Table 1:** Effect of sample size, from 20 to 60 individuals, on genetic diversity parameters calculated using individual sequencing (ind-seq) data. The parameters analyzed include the number of single nucleotide polymorphisms (SNPs), observed heterozygosity (H_O_), expected heterozygosity (H_E_), inbreeding coefficient (F_IS_), and allelic richness (Ae), along with their minimum and maximum (min-max) values. The “Significance” row indicates the statistical significance of these effects, as determined by Tukey’s test, where “ns” represents non-significant differences, while “**” indicates statistically significant differences at p < 0.01.

Sample size	Number of SNPs	Observed heterozygosity (H_O_)	Expected heterozygosity (H_E_)	Inbreeding Coefficient (F_IS_)	Allelic richness (Ae) (min -max)
20	2494	0.148	0.181	0.205	1.611 (1.568-1.672)
30	2698	0.148	0.183	0.206	1.657 (1.612-1.729)
40	2696	0.150	0.185	0.202	1.691 (1.632-1.771)
50	2800	0.151	0.188	0.204	1.734 (1.682-1.81)
60	2863	0.151	0.187	0.201	1.750 (1.703-1.826)
Significance	ns	ns	ns	ns	**

#### 3.2.2 Effect of sample size on population structure.

The AMOVA results indicated significant genetic variation among accessions (p < 0.0001), explaining 13% of the total genetic diversity, with the remaining 87% attributed to variability within accessions. Detailed AMOVA results for each sample size are provided in Table H of S1 Appendix.

### 3.3 Diversity and population structure analysis with pool-seq datasets

#### 3.3.1 Effect of sample size and sequencing depth on genetic diversity parameters.

**Fig 7:**
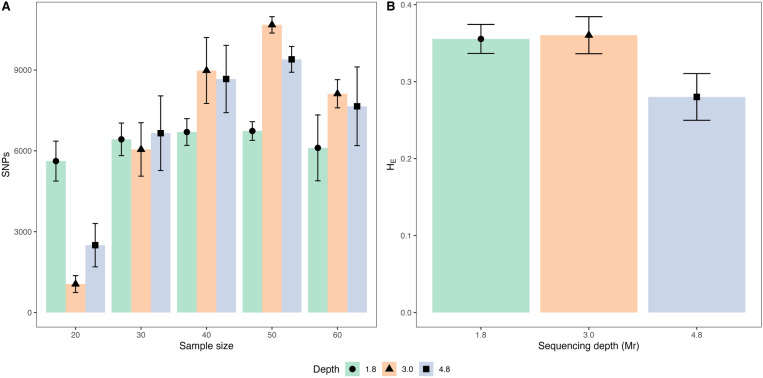
Effect of sample size and sequencing depth on single nucleotide polymorphisms (SNP) number and the influence of sequencing depth on Expected heterozygosity (H_E_) with pool-seq dataset. Panel (A.) presents the average number of SNPs (SNPs) across accessions, along with the corresponding standard deviation, stratified by sample size and sequencing depth. Panel (B.) illustrates the average expected heterozygosity and its standard deviation across all accessions at varying sequencing depths.

Sample size and sequencing depth were observed to affect the number of SNPs detected (F (8,60) = 17.7, p < 0.001; see Table B in S2 Appendix for details). Specifically, pools of 50 individuals consistently yielded more SNPs compared than pools of 20, 30, and 60 individuals at both 3.0 Mr and 4.8 Mr sequencing depths (F (4,60) = 84.4, 45, respectively, p < 0.05). For pools of 20 individuals, the number of SNPs varied significantly across the three sequencing depths (F (2,60) = 33.4, p < 0.05). In contrast, for larger sample sizes (40, 50, and 60 individuals), significant differences in SNPs count were only observed between the 1.8 Mr and the higher sequencing depths, 3.0 and 4.8 Mr (F (2,60) = 9.4, 24.8, 6.8, respectively, p < 0.05), being the largest average number of SNPs achieved at the 3.0 Mr sequencing depth (Fig 7A). In contrast to the observed trend for the SNP number, the average H_E_ significantly decreased at the 4.8 Mr sequencing depth compared to 1.8 and 3 Mr depths (F (2,12) = 30.3, 45, p < 0.001; Fig 7B). However, H_E_ did not exhibit any significant differences across varying sample sizes (see Table I in S2 Appendix for further details). Detailed data on the number of SNPs and H_E_ for each accession, across the five sample sizes (20, 30, 40, 50, 60) and three sequencing depths (1.8, 3.0, 4.8 Mr) are summarized in Table A of the S2 Appendix.

#### 3.3.2 Effect of sample size and sequencing depth on population structure.

The AMOVAs test revealed significant genetic differentiation among accessions; however, most of the total genetic variation, from 73% to 88%, was attributed to diversity within accessions. The proportion of within-accession variation varied depending on the combination of sample size and sequencing depth (see Table J in S2 Appendix).

### 3.4 Comparison of genetic diversity and population structure obtained with ind-seq and pool-seq data

#### 3.4.1 Sample size and sequencing depth effect on expected heterozygosity estimated from ind-seq and pool-seq data.

**Fig 8:**
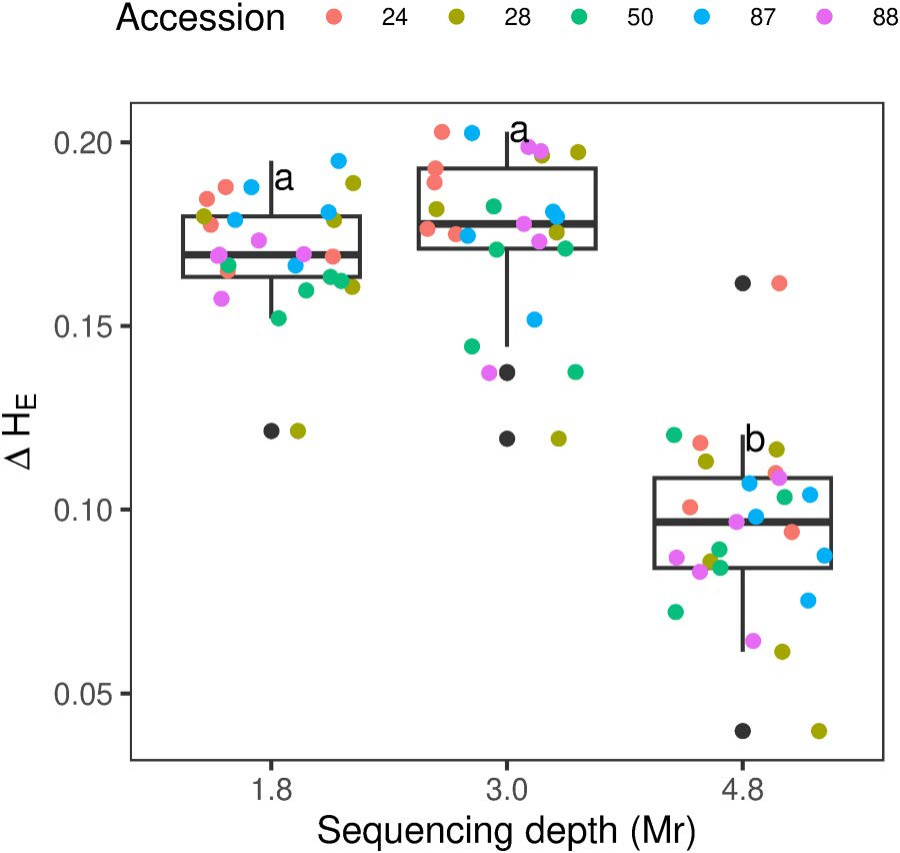
Boxplot illustrating influence of sequencing depth in ΔH_E_: H_E_ pool-seq – H_E_ ind-seq. Each dot represents a single accession, with color explained in the legend. The line inside each box represents the median, while the lower and upper box edges indicate the first and third quartiles, respectively. The whiskers extend from the box to the minimum and maximum values within 1.5 times the interquartile range from the first and third quartiles, respectively. Tukey’s test (p < 0.05) is indicated by letters statistical differences between groups. Plots were generated using the package “ggplot2” in R [53].

The H_E_ estimated for pool-seq were consistently higher than those for ind-seq. A major convergence in H_E_ estimates from both methods was observed at a sequencing depth of 4.8 Mr. This convergence resulted in a statistically significant reduction in ΔH_E_ values (F (2,12) = 92.8, p < 0.001), with a median lower than 0.1 (Fig 8; see more in Table A in S3 Appendix).

#### 3.4.2 Evaluation of population structure: ind-seq vs. pool-seq.

Both ind-seq and pool-seq indicated comparable levels of substantial intra-accession genetic diversity across the evaluated accessions (Table 2). However, the F_IS_ values revealed ongoing inbreeding processes in all accessions. Notably, pool-seq data exhibited higher genetic diversity based on H_E_ values compared to ind-seq, although Ae values were consistently lower. Accession 50 displayed the highest H_O_ and H_E_ values, alongside the lowest F_IS_ in ind-seq, and exhibited the highest H_E_ and Ae in pool-seq. Contrastingly, based on ind-seq data accession 28 showed the lowest H_O_, H_E_, and Ae values, while accession 24 had the highest F_IS_ value observed in this study. In pool-seq, accessions 88 and 87 exhibited the lowest H_E_ and Ae values, respectively.

**Table 2:** Comparative population genetics parameters for each accession using a sample size of 50 individuals, analyzed with individual sequencing (ind-seq) and pooled sequencing (pool-seq). The table includes observed data for heterozygosity (H_O_), expected heterozygosity (H_E_), inbreeding coefficient (F_IS_), and allelic richness (Ae) from Ind-seq, while pool-seq data includes H_E_ and Ae. Averages across all accessions are also presented.

	Ind-seq				Pool-seq	
Accession	H_O_	H_E_	F_IS_	Ae	H_E_	Ae
24	0.14	0.19	0.27	1.71	0.28	1.50
28	0.13	0.17	0.21	1.68	0.28	1.47
50	0.18	0.21	0.17	1.71	0.29	1.55
87	0.15	0.18	0.19	1.75	0.28	1.46
88	0.16	0.19	0.18	1.81	0.27	1.47
Average	0.15	0.19	0.20	1.73	0.28	1.49

#### 3.4.3 Comparative analysis of population structure: ind-seq vs. pool-seq.

AMOVA analysis of both ind-seq and pool-seq data indicated statistically significant population structure. In both methods, within-accession diversity accounted for most of the total variation, explaining 87% in ind-seq and 75% in pool-seq. In contrast, between-accession variation explained 13% and 25% of the total diversity in ind-seq and pool-seq, respectively (Table 3).

**Table 3:** Analysis of molecular variance (AMOVA) was conducted on a sample size of 50 individuals to assess genetic variation within and between accessions using both individual sequencing (ind-seq) and pooled sequencing (pool-seq) approaches. The table presents the degrees of freedom and percentage of variation for each source, with *p-value* reported for variation between-accession.

	Ind-seq			Pool-seq		
	Degrees of freedom	% Variation	p-value	Degrees of freedom	% Variation	p-value
Between accessions	4	13	1x10^-04^	4	25	1x10^-04^
Within accessions	240	87	–	15	75	–
Total	244	100	–	19	100	–

Pairwise genetic distances, calculated using both Nei’s distance and F_ST_, revealed strong correlations and highlighted the genetic differentiation among accessions. The Mantel test, applied to Nei’s genetic distance matrices derived from both ind-seq and pool-seq data, revealed a strong and statistically significant correlation between the two matrices (r = 0.991, p-value < 0.05; S7 Table). Pairwise F_ST_ analysis of the ind-seq data indicated moderate to high genetic differentiation between nearly all accessions (Table 4). Accessions from the same ecoregion exhibited the highest degree of genetic similarity, as observed for the accessions 28 and 87 from Gondwanic sediments; in contrast, the highest F_ST_ value was observed between accessions 28 from the Godwanic sediments region and accession 50, collected in the Crystalline Shield region.

**Table 4:** Pairwise fixation index (F_ST_) values calculated between accessions using individual sequencing (ind-seq) dataset, based on a sample size of 50 individuals.

Accession	24	28	50	87
28	0.182			
50	0.196	0.237		
87	0.178	0.061	0.223	
88	0.125	0.124	0.165	0.107

**Fig 9:**
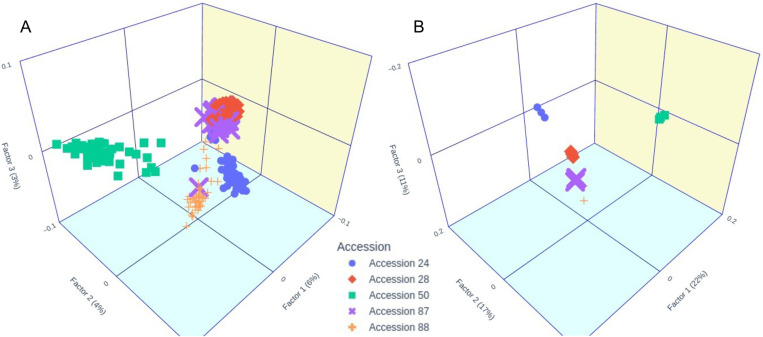
Multidimensional scaling (MDS) based on Roger’s distance. (A.) MDS plot derived from individual sequencing (ind-seq) data using 50 individuals per accession and 2,124 single nucleotide polymorphisms (SNPs). (B.) MDS plot derived from pool sequencing (pool-seq) data using pools of 40, 50, and 60 plants with a sequencing depth of 4.8 Mr and 63,017 SNPs. Graphs were generated using the “plotly” library in Python [71].

The three-dimensional MDS plots, generated from both ind-seq and pool-seq data, revealed four distinct groups with a similar distribution pattern (Fig 9A and 9B). The first group predominantly consists of accessions collected from the Gondwanic sediments, mainly accessions 28 and 87, along with a few individuals from accessions 24 and 88, which are from Graven Merín and Graven Santa Lucía, respectively. The second group is primarily composed of accession 88, with one individual from accession 87 and another from accession 24 also included in the ind-seq MDS. Accession 50, from the Crystalline Shield sediments, clustered into a distinct third group. Finally, the remaining individuals from accession 24 (Graven Merin) formed a clearly separate fourth group.

## 4. Discussion

### 4.1 Comparison of SNPs and allele frequencies obtained with individual and pooled samples

To achieve high concordance between allele frequencies estimates from individual and pooled sequencing data – measured by the Concordance Correlation Coefficient (CCC)- a minimum sample size of 30 individuals was required. Increasing the sample size to 50 plants further enhanced the median number of SNPs detected in pooled samples, Representativity and CCC. These findings are consistent with previous reports in diploid crops, such as *Lolium perenne*, where a strong concordance between individual and pooled datasets was observed using 40 plants [23]. Similarly, other research has recommended pooling at least 40 individuals to minimize variability in DNA contribution from each individual [42]. Additionally, in a predominantly outcrossing diploid species *Arabidopsis lyrata*, pools of 25 plants exhibited a higher correlation with individual sequencing compared to pools of 14 individuals [21]. In contrast, another study suggested that the pooled sequencing of 5 and 10 individuals might be enough to provide comparable insights to those from sequencing a single plant in a self-pollinating species such as *Oryza barthii* A. Chev., *O. glaberrima* Steud., and *O. sativa* L. [20]. These studies highlight the importance of selecting an appropriate number of individuals to ensure that the pooled samples adequately represent the genetic diversity of the population. Our results provide further evidence of this approach.

The correlation between allele frequency estimates from individual and pooled sequencing datasets declined as the missing data (MD) threshold increased, as observed by previous studies [23]. MD threshold is a critical factor in population genomics research, significantly affecting the confidence of allele frequency calculations and the preservation of loci for subsequent analyses [29,30]. In accordance with the results described for *Lolium perenne* [23], we applied a 10% MD threshold, focused on maximizing the accuracy of the allele frequency estimation within each accession. Conventionally, studies on diversity have employed higher and arbitrary MD thresholds [30], which has increased the number of loci retained for analysis and preserved rare or specific population variants. Concurrently, the implementation of a relaxed threshold criterion was found to introduce noise, thereby compromising data reliability. It is crucial to note that the optimal MD threshold is context-dependent and influenced by multiple factors. In the context of this study, key considerations include the genetic diversity present within *B. auleticus*, the specific research objectives, and the intended downstream analyses. Therefore, determining the most appropriate maximum MD threshold requires an empirical approach tailored to each *B. auleticus* dataset. This ensures a balance trade-off between data quality and genome-wide coverage [29,30].

Maximum concordance among allele frequency estimates in individuals and pools was observed at the highest sequencing depth (4.8 Mr). It is noteworthy that this did not result in any substantial variation in the proportion of shared SNPs (Representativity), or the number of SNPs detected in pooled samples compared to the 3.0 Mr sequencing depth. The increase on CCC can be attributed to the sequencing of multiple libraries from the same pool, a strategy employed before with *Lolium perenne* pools [23]. Given the high degree of genetic diversity inherent in DNA pools, mechanical mixing during pipetting may introduce random fluctuations in the proportions of alleles during the library construction. Additionally, biases during the library preparation could potentially incorporate an overrepresentation of certain fragments while causing stochastic depletion of others [72]. To minimize these biases, a previous study employed a successful strategy of sequencing the same pool multiple times and consolidating the results [23]. Although increasing the sequencing depth from 1.8 to 3.0 Mr contributed to the detection of greater number of SNPs (Fig 7A), neither Representativity nor CCC experienced significant changes. This finding is consistent with the existing literature, suggesting that the benefits of increased sequencing depth may reach a plateau beyond a certain threshold [21,28,73]. Our results emphasize the importance of optimizing both sequencing depth and the number of replicates to achieve a more accurate representation of genetic diversity.

We observed a slight, non-significant increase in Representativity and CCC when the MAF threshold was reduced from 0.01 to 0.05 in pools. This result suggests that reducing the MAF cut-off could improve the detection of alleles at low frequencies and may mitigate allelic dropout previously observed with pool-seq [28]. While distinguishing true low-frequency variants from sequencing errors is a known limitation of pool-seq [42], the DArTseq® SNP calling algorithm used in the present research moderates this issue. This algorithm was designed to minimize errors through applying stringent quality control measures, including reproducibility checks utilizing internal technical controls (see Section 2.2).

The optimization of parameters such as sample size, MD, sequencing depth, and MAF enhanced the shared SNP proportion and allele frequency concordance between individual and pooled sequencing datasets. Representativeness averaged approximately 65%, while CCC surpassing 0.9. Based on our empirical evaluation, we suggest pooling samples with a minimum of 30 individuals, using a sequencing depth of 4.8 Mr, a maximum MD threshold of 10%, and a minimum MAF of 0.01. The present findings provide substantial support for the hypothesis of this study and validate the use of pooled samples for reliable allele frequency estimation in *B. auleticus*.

### 4.2 Effect of sample size on the genetic diversity and population structure analysis with ind-seq dataset

Our results indicate that a sample size of 20 individuals per population of *B. auleticus* is adequate for population structure analysis, however, allelic richness (Ae) only stabilizes when the sample size reaches at least 30 individuals per population. This finding is consistent with previous research, which shows that heterozygosity is relatively independent to sample size variation, whereas Ae is highly sensitive on it [74]. In contrast, these results differ from findings reported in two studies conducted on diploid, outcrossing species. For instance, in *Amphirrhox longifolia*, a sample size of more than eight individuals was sufficient to obtain reliable estimates of genetic diversity metrics (Ae, H_O_, and H_E_) when using a minimum of 1,000 SNPs markers [25]. A similar conclusion was reached in research on *Zea mays ssp. parviglumis* and *Zea mays ssp. mexicana*, where six and nine individuals were enough to accurately estimate H_E_ and F_IS_, respectively [27]. Additionally, precise estimates of F_ST_ can be achieved with as few as two individuals, when enough SNPs (≥1,500) are utilized in *A. longifolia* and. *Zea mays* [25,27]. These findings highlight the critical role of balancing sample size and marker density to ensure the robustness of population genetic analyses. Our findings align with previous studies, further emphasize the importance of considering specific research objectives when determining the optimal sample size for population genetic studies [25,38,75].

The population structure observed using our data is consistent with results reported for other allogamous polyploid grasses. The F_IS_ values calculated for *B. auleticus* here are similar to those obtained for the allotetraploid *Phalaris aquatica* L. (F_IS_ = 0.18) and the autotetraploid *Dactylis glomerata* L. (F_IS_ = 0.18), but considerably higher than the F_IS_ value calculated for the allohexaploid *Festuca arundinacea* Schreb (F_IS_ = 0.025) [76,77]. Conversely, *P. aquatica* exhibited lower observed (H_O_ = 0.1) and expected (H_E _= 0.14) heterozygosity than *B. auleticus* [77]. The AMOVA results indicate that the diversity patterns in *B. auleticus* resemble those found in *P. aquatica*, *D. glomerata*, and *F. arundinacea*, where most of the genetic variation resides at the within-population level [76,77].

### 4.3 Effect of sample size and sequencing depth on genetic diversity and population structure analysis with pool-seq dataset

Consistent with previous findings, the number of detected SNPs increased with both larger sample sizes and higher sequencing depth [21,28]. However, in the present study, SNP detection reached a plateau at a sample size of 40 and a sequence depth of 3.0 Mr, suggesting a saturation point beyond which additional sequencing effort yielded minimal gains. This indicates a potential upper limit in the number of detectable SNPs within the five *B. auleticus* accessions analyzed, reflecting the extent of genetic diversity present in the sampled population.

The observed decline in H_E_ at a sequencing depth of 4.8 Mr suggests an overestimation of this parameter at lower sequencing depths. This finding highlights the importance of optimizing sequencing depth in population genetic studies, as overestimated population parameters may lead to misleading conclusions [30]. Notably, the effect of sequencing depth on H_E_ in pooled samples appears to surpass the effect of sample size, emphasizing its critical role in experimental design.

### 4.4 Comparison of diversity and population structure analysis between ind-seq and pool-seq datasets

As previously reported, the pool-seq detected more SNPs than the ind-seq method [21,23]. In this study the discrepancy can be attributed to two factors: the higher sequencing depth and the more relaxed MAF threshold in pool-seq. The sequencing depth in pool-seq was 5.3 times higher than in ind-seq, resulting from the combination of both reads tissue and library replicates.

At all sequencing depths evaluated, the H_E_ values obtained from pool-seq were higher than those from ind-seq, with the greatest convergence observed at a pool-seq sequencing depth of 4.8 Mr. This discrepancy might be attributable to three main factors. First, ind-seq relies on binary presence/absence data (0 or 1), while pool-seq uses allele counts. This continuous scale may be particularly advantageous for polyploid species such as *B. auleticus*. Second, the MAF threshold was more relaxed in the pool-seq, increasing the proportion of loci with high H_E_. Third, the software used for pool-seq applies data imputation techniques to address missing data absent values, unlike the ind-seq software [64,66].

Although pooled samples exhibit higher expected heterozygosity than individual samples, they showed lower Ae. This incongruity may be attributed to the computational procedure. The H_E_ estimation summed allele counts across all samples of an accession, while the Ae estimation did not. This difference in calculation procedure may affect the detection of rare alleles and contribute to the observed divergences.

A high inbreeding coefficient was observed in the accessions of *Bromus auleticus* analyzed here. This inbreeding pattern may reflect population fragmentation, often exacerbated by agricultural practices and overgrazing [1,78]. Also, the perennial nature of *B. auleticus* could contribute to inbreeding by facilitating more frequent mating among related individuals within small and isolated populations. Although this species is considered predominantly outcrossing, the reduced genetic exchange in fragmented populations could still contribute to the observed inbreeding levels. Furthermore, self-incompatibility mechanisms of *B. auleticus* postulated in previous studies [9,79–81], is influenced by the species mating system and heterozygosity patterns, further affecting reproductivity dynamics and genetic diversity.

Accession 50 exhibited the highest H_O_, H_E_, and lower F_IS_ in ind-seq analyses, and the highest H_E_ and Ae in pool-seq, indicating strong consistency between these two approaches. Despite some discrepancies in the rankings of the least diverse accessions between methodologies, the overall conclusions remain aligned. This reliably is supported by the similar diversity estimates obtained for all accessions across both sequencing strategies.

In this study, pools of 50 plants yielded the highest number of SNPs and the lowest ΔH_E_ values, indicating improved accuracy and resolution in diversity estimates. At 4.8Mr, SNPs counts for these larger pools were significantly higher than those observed in pools of 20 and 30 individuals. Moreover, pools of 50 individuals showed the highest CCC, further supporting the effectiveness of this pool size. It is important to note that the CCC only includes SNPs shared between ind-seq and pool-seq, whereas ΔH_E_ estimates included all SNPs detected by each methodology independently. Consequently, the low ΔH_E_ (mean 0.09 across all accessions) for the 50 plants highlights the efficiency of pool-seq in analyzing intra-accession diversity in *B. auleticus*.

Population structure estimates from both ind-seq and pool-seq datasets exhibited a high degree of consistency. Both AMOVAs revealed significant divergence among accessions, with a predominance of intraspecific diversity. This intraspecific diversity was further supported by the MDS analysis of the ind-seq dataset. Additionally, genetic distance measurements obtained using the paired F_ST_ were consistent with findings from other studies of the *Bromus* genus [47]. The fixation index and Nei’s distance revealed differences among ecoregions congruent with previous phenotypic analyses, suggesting the possible presence of ecotypes associated with each ecoregion [6]. However, additional analyses with more populations are needed to confirm this hypothesis.

The genetic structure of *B. auleticus,* as determined by both the ind-seq and pool-seq methods is consistent with the genetic structure of the alogamous species [82]. These findings are supported by previous studies on this grass [6,83–86]. Thus, the results of this study reinforce the validity of the pool-seq as a reliable and effective method for analyzing the genetic structure of *B. auleticus* populations.

### 4.5 Proposed workflows

**Fig 10:**
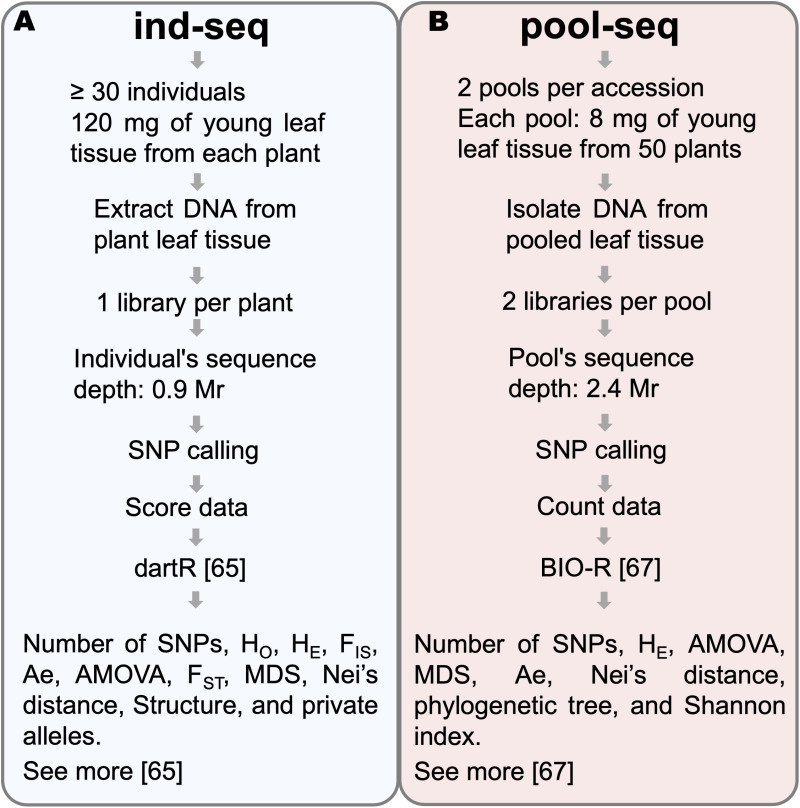
Proposed workflows for analyzing genetic diversity in *B. auleticus.* (A.) Ind-seq: individual sequencing approach. (B.) pool-seq: pooled sequencing workflow. The diagram outlines the number of samples, sequencing depth, R packages used, and the analyses proposed for each workflow. This diagram was created using Microsoft PowerPoint.

Fig 10 presents two alternatives workflows proposed in this study for assessing genetic diversity in *B. auleticus*: A) individual sequencing (ind-seq) and B) pooled sequencing (pool-seq). The selection of the most appropriate workflow depends on the research objectives and the biological characteristics of the species. Factors such as target population(s), sampling design, and bioinformatic tools selection are influenced by these considerations [75,87].

A key difference between the two workflows is their sampling demand. Although ind-seq requires approximately 1.7 times fewer plants than pool-seq, it demands 15-fold more leaf tissue per plant. This make pool-seq particularly advantageous for slow-growing species such as *B. auleticus* [2], as it minimizes the time required for genotyping.

Furthermore, pool-seq offers advantages in sample handling, data management, and cost-efficiency. While ind-seq typically processes 30 samples per accession, pool-seq reduces this to four, a 7.5-fold decrease. This reduction may minimize sample tracking errors, lower library sequencing costs, shorten processing time, and decrease total data volume by 5.6-fold. In this study, the pool-seq workflow cost was 5.5 times lower than that of ind-seq. This finding aligns with the reported outcomes of previous studies [20,28,42,87], reinforcing the economic and logistical benefits of pooled sequencing in appropriate contexts.

The bioinformatics tools used in this study were user-friendly and required relatively modest computational resources. DArTseq data can be analyzed on standard desktop computers using software such dartR [64] and/or Bio-R [66]. In contrast, the analysis of data obtained by other high-throughput methods may require higher computational capacity, including the use of dedicated servers. Notably, dartR is designed for the analysis of score data, whereas Bio-R supports both score and count data and offers a more intuitive interface, enhancing its usability for a broader range of users.

## 5. Conclusions

*B. auleticus* is a native winter forage species of the Río de la Plata Grasslands, a recognized center of grass diversity. Given the ongoing regional grasslands loss, the conservation and sustainable use of *B. auleticus* is imperative. This research compared two methods for assessing genetic diversity, individual sequencing (ind-seq) and pooled sequencing (pool-seq), across five *B. auleticus* accessions, and found consistent results between them.

Although, each method has distinct strengths and limitations, making them suitable for different applications. The ind-seq method provides high-resolution data and is well-suited for applications requiring fine-scale genetic information, such as parentage verification and detection of rare alleles. However, is broader implementation is often limited by high costs, grater labor demands, and increased computational requirements, making it more feasible for small-scale studies. Theoretically, ind-seq introduces minimal bias and enables accurate estimates of allelic diversity and heterozygosity.

In contrast, pool-seq offers a cost-effective and time-efficient alternative, especially suitable for large-scale genetic studies. It is particularly useful in landscape genomics and in identifying populations for breeding and conservation (*in-situ* and *ex-situ*). Despite limitation in rare allele detection, pool-seq reliably effectively estimates allele frequencies and population structure.

Ultimately, the selection between ind-seq and pool-seq should be define by the specific research objectives, available resources, required genetic resolution, and cost-efficiency balance. Both approaches provide valuable insights to support the conservation and genetic improvement of *B. auleticus*, offering complementary tools for advancing its sustainable use.

## Supporting information

S1 Table Passport data and phenotypic characterization of sequenced accessions. This table provides information of the sequenced accessions, including their germplasm bank ID, geographic coordinates (longitude and latitude) of collection, geological formation of the collection site, and associated phenotypic characteristics. https://doi.org/10.6084/m9.figshare.28225535.v1.
(XLSX)

S2 Table SNP calling score from individual and pooled sequencing data. Matrix for SNP analysis in *Bromus auleticus* genotypes, detailing metrics for individual and pooled data sets. It includes 130,261 markers with their allele identification, call rates, homozygosity and heterozygosity frequencies, polymorphism information content (PIC), and data reproducibility. The table contains 296 samples, comprising 58 individuals samples from accessions 50 and 87, and 60 individual samples from accessions 24, 28 and 88. The other 100 samples represent pooled data, 20 per accession. https://doi.org/10.6084/m9.figshare.28225493.v1.
(ZIP)

S3 Table SNP calling counts from individual and pool sequencing data. Comprehensive count matrix for SNP analysis in *Bromus auleticus* genotypes, detailing metrics for both individual and pooled data sets. It includes 130,261 markers with their allele identification, call rates, homozygosity and heterozygosity frequencies, polymorphism information content (PIC), and reproducibility. The table contains 296 samples, consisting of 58 individual samples from accessions 50 and 87, and 60 individual samples from the remaining accessions. The remaining 100 samples represent pooled data, 20 for each accession. https://doi.org/10.6084/m9.figshare.28225550.v1.
(ZIP)

S4 Table SNP calling score from individual sequencing data. Scoring matrix for SNP analysis in *Bromus auleticus* genotypes, detailing metrics for both individual and pooled data sets. It includes 156,801 markers with their respective allele identifications, call rates, homozygosity and heterozygosity frequencies, polymorphism information content (PIC), and reproducibility metrics. The table contains 196 samples, consisting of 58 individual samples from accessions 50 and 87, and 60 individual samples from the remaining accessions. https://doi.org/10.6084/m9.figshare.28225544.v2.
(ZIP)

S5 Table SNP calling counts from pooled sequencing data. Comprehensive count matrix for SNP analysis in *Bromus auleticus* genotypes, detailing metrics for both individual and pooled data sets. The matrix includes 130,261 markers with their respective allele identifications, call rates, homozygosity and heterozygosity frequencies, polymorphism information content (PIC), and reproducibility. The table contains 296 samples, consisting of 58 individual samples from accessions 50 and 87, and 60 individual samples from the remaining accessions. The remaining 100 samples represent pooled data, with 20 samples allocated for each accession. https://doi.org/10.6084/m9.figshare.28225559.v1.
(ZIP)

S6 Table Comparison of allele frequencies: Individuals versus pooled datasets. Representativity, concordance correlation coefficient (CCC), and number of shared SNPs, including confidence interval calculations, derived from frequency comparisons of individual and pooled datasets for each accession. The metrics are analyzed concerning sample size, missing data, sequence depth, and minor allele frequency (MAF) pools. https://doi.org/10.6084/m9.figshare.28225520.v1.
(CSV)

S7 Table Nei’s genetic distances calculation from ind-seq and pool-seq data. Nei’s genetic distances between the five accessions calculated from individual sequencing (ind-seq; left) and pooled sequencing (pool-seq; right) data. A sample size of 50 was employed in both analyses. https://doi.org/10.6084/m9.figshare.28225511.v1.
(XLSX)

S1 Appendix Effect of sample size on diversity and population structure analysis of *Bromus auleticus* employing ind-seq dataset. This appendix provides tables summarizing genetic diversity parameters -number of single nucleotide polymorphism (SNP), observed heterozygosity (H_O_), expected heterozygosity (H_E_), inbreeding coefficient (F_IS_), and allele richness (Ae), analyses of variance and post-hoc comparisons of each parameter, and analysis of molecular variance (AMOVA) results across varying sample sizes. https://doi.org/10.6084/m9.figshare.28225490.v2.
(DOCX)

S2 Appendix Effects of sample size and sequencing depth on diversity and population structure analysis of *Bromus auleticus* with pool-seq dataset. This document provides tables containing population genetic metrics -number of single nucleotide polymorphism (SNP) and expected heterozygosity (H_E_)-, two-way ANOVA, and AMOVA results across the five accessions, sample sizes, and sequencing depths. https://doi.org/10.6084/m9.figshare.28225532.v1.
(DOCX)

S3 Appendix Comparison of accession diversity between ind-seq and pool-seq datasets. This appendix provides tables summarising ΔH_E_ values across the five accessions, sample sizes and sequencing depths, and the associated ANOVAs. https://doi.org/10.6084/m9.figshare.28225553.v1.
(DOCX)
