# Supplementary material for: Optimizing genomic diversity assessments for conservation of Bromus auleticus (Trinius ex Nees) using individual and pooled sequencing
Source: PLoS One. 2025 Jun 25;20(6):e0325548. doi: 10.1371/journal.pone.0325548 (PMC12194279; doi:10.1371/journal.pone.0325548)
Supplement: S1 Appendix — This appendix provides tables summarizing genetic diversity parameters -number of single nucleotide polymorphism (SNP), observed heterozygosity (HO), expected heterozygosity (HE), inbreeding coefficient (FIS), and allele richness (Ae), analyses of variance and post-hoc comparisons of each parameter, and analysis of molecular variance (AMOVA) results across varying sample sizes. https://doi.org/10.6084/m9.figshare.28225490.v2. (DOCX) [file pone.0325548.s008.docx]

**Effect of sample size on diversity and population structure analysis of *Bromus auleticus* employing ind-seq dataset**

Table A: Genetic diversity parameters across five Bromus auleticus accessions at a sequencing depth of 0.9 million reads per individual and minimum allele frequency (MAF) of 0.05. Metrics include observed heterozygosity (H_O_), expected heterozygosity (H_E_), inbreeding coefficient (F_IS_), and allele richness (Ae).

| **Accesion** | **Sample size** | **SNPs** | **H_O_** | **H_E_** | **F_IS_** | **Ae** |
| --- | --- | --- | --- | --- | --- | --- |
| 24 | 20 | 2303 | 0.139 | 0.185 | 0.270 | 1.577 |
| 28 | 20 | 2340 | 0.128 | 0.158 | 0.211 | 1.568 |
| 50 | 20 | 2517 | 0.172 | 0.208 | 0.194 | 1.633 |
| 87 | 20 | 2511 | 0.147 | 0.173 | 0.171 | 1.608 |
| 88 | 20 | 2798 | 0.152 | 0.181 | 0.178 | 1.672 |
| 24 | 30 | 2475 | 0.139 | 0.187 | 0.269 | 1.612 |
| 28 | 30 | 2529 | 0.130 | 0.162 | 0.210 | 1.612 |
| 50 | 30 | 2621 | 0.172 | 0.207 | 0.183 | 1.654 |
| 87 | 30 | 2835 | 0.146 | 0.176 | 0.186 | 1.679 |
| 88 | 30 | 3030 | 0.151 | 0.182 | 0.183 | 1.729 |
| 24 | 40 | 2436 | 0.137 | 0.185 | 0.268 | 1.632 |
| 28 | 40 | 2548 | 0.131 | 0.165 | 0.221 | 1.649 |
| 50 | 40 | 2639 | 0.178 | 0.210 | 0.164 | 1.688 |
| 87 | 40 | 2836 | 0.147 | 0.179 | 0.188 | 1.718 |
| 88 | 40 | 3020 | 0.158 | 0.187 | 0.170 | 1.771 |
| 24 | 50 | 2775 | 0.138 | 0.186 | 0.267 | 1.714 |
| 28 | 50 | 2602 | 0.133 | 0.167 | 0.213 | 1.682 |
| 50 | 50 | 2672 | 0.179 | 0.214 | 0.172 | 1.713 |
| 87 | 50 | 2878 | 0.149 | 0.182 | 0.190 | 1.751 |
| 88 | 50 | 3071 | 0.158 | 0.191 | 0.180 | 1.810 |
| 24 | 60 | 2854 | 0.137 | 0.184 | 0.260 | 1.728 |
| 28 | 60 | 2679 | 0.134 | 0.168 | 0.212 | 1.703 |
| 50 | 60 | 2713 | 0.178 | 0.214 | 0.177 | 1.725 |
| 87 | 60 | 2936 | 0.152 | 0.182 | 0.173 | 1.767 |
| 88 | 60 | 3133 | 0.156 | 0.190 | 0.183 | 1.826 |

Table B: Results of one-way ANOVA examining the influence of sample size on the number of single nucleotide polymorphisms (SNPs) identified.

|  | **Df** | **Sum Sq** | **Mean Sq** | **F** | **value** | **Pr(>F)** |
| --- | --- | --- | --- | --- | --- | --- |
| **Sample size** | 4 | 392626 | 98157 | 2.298 | 0.0946 | - |
| **Residuals** | 20 | 854375 | 42719 |  |  |  |

Table C: One-way ANOVA results assessing the impact of sample size on observed heterozygosity (H_O_) across accessions.

|  | **Df** | **Sum Sq** | **Mean Sq** | **F** | **value** | **Pr(>F)** |
| --- | --- | --- | --- | --- | --- | --- |
| **Sample size** | 4 | 0.000069 | 1.724E-05 | 0.057 | 0.993 | - |
| **Residuals** | 20 | 0.006060 | 3.03E-04 |  |  |  |

Table D: One-way ANOVA results showing the effect of sample size on expected heterozygosity (H_E_) across accessions.

|  | **Df** | **Sum Sq** | **Mean Sq** | **F** | **value** | **Pr(>F)** |
| --- | --- | --- | --- | --- | --- | --- |
| **Sample size** | 4 | 0.000189 | 4.735E-05 | 0.164 | 0.954 | - |
| **Residuals** | 20 | 0.005762 | 2.881E-04 |  |  |  |

Table E: One-way ANOVA results showing the influence of sample size on the Inbreeding Coefficient (F_IS_) across accessions.

|  | **Df** | **Sum Sq** | **Mean Sq** | **F** | **value** | **Pr(>F)** |
| --- | --- | --- | --- | --- | --- | --- |
| **Tamaño_muestra** | 4 | 0.000088 | 0.000022 | 0.015 | 1 | - |
| **Residuals** | 20 | 0.030075 | 0.001504 |  |  |  |

Table F: One-way ANOVA results showing the effect of sample size on allele richness (Ae) across accessions, highlighting statistically significant outcomes.

|  | **Df** | **Sum Sq** | **Mean Sq** | **F** | **value** | **Pr(>F)** |
| --- | --- | --- | --- | --- | --- | --- |
| **Sample size** | 4 | 0.06367 | 0.015918 | 6.608 | 0.00147 | ** |
| **Residuals** | 20 | 0.04818 | 0.002409 |  |  |  |
| **Signif. codes:** | 0 ‘***’ 0.001 ‘**’ 0.01 ‘*’ 0.05 ‘.’ 0.1 ‘’ 1 | | | | | |

Table G: Tukey's post-hoc pairwise comparisons for allele richness (Ae), illustrating differences among sample size groups.

|  | **diff** | **lwr** | **upr** | **p adj** |
| --- | --- | --- | --- | --- |
| **30-20** | 0.04604686 | -0.0468418522 | 0.1389356 | 0.584226691 |
| **40-20** | 0.08001716 | -0.0128715504 | 0.1729059 | 0.112908095 |
| **50-20** | 0.12260479 | 0.0297160799 | 0.2154935 | 0.006307575 |
| **60-20** | 0.13831187 | 0.0454231564 | 0.2312006 | 0.002016013 |
| **40-30** | 0.03397030 | -0.0589184092 | 0.1268590 | 0.807355476 |
| **50-30** | 0.07655793 | -0.0163307789 | 0.1694466 | 0.138647318 |
| **60-30** | 0.09226501 | -0.0006237024 | 0.1851537 | 0.052094488 |
| **50-40** | 0.04258763 | -0.0503010808 | 0.1354763 | 0.651523452 |
| **60-40** | 0.05829471 | -0.0345940043 | 0.1511834 | 0.360200976 |
| **60-50** | 0.01570708 | -0.0771816346 | 0.1085958 | 0.985808956 |

Table H: Analysis of molecular variance (AMOVA) results for varying sample sizes, partitioning genetic variation within and between groups. Statistical metrics include degree of freedom (DF), sum of squares (SumOfSqs), mean squares (MS), population differentiation statistics (Phi), Coefficient of Variance (Sigma), and percent of the total variance explained by each source of variance (PercVar).

|  | DF | SumOfSqs | MS | Phi | p-value | Sigma | PercVar |
| --- | --- | --- | --- | --- | --- | --- | --- |
| **Simple size: 20** | | | | | | | |
| **Between Groups** | 4 | 0.535 | 0.134 | 0.130 | 0.000 | 0.005 | 13 |
| **Within Groups** | 94 | 3.173 | 0.034 | - | - | 0.034 | 87 |
| **Total** | 98 | 3.708 | 0.038 | - | - | 0.039 | 100 |
| **Simple size: 30** | | | | | | | |
| **Between Groups** | 4 | 0.740 | 0.185 | 0.132 | 0.000 | 0.005 | 13 |
| **Within Groups** | 142 | 4.809 | 0.034 | - | - | 0.034 | 87 |
| **Total** | 146 | 5.549 | 0.038 | - | - | 0.039 | 100 |
| **Simple size: 40** | | | | | | | |
| **Between Groups** | 4 | 0.928 | 0.232 | 0.131 | 0.000 | 0.005 | 13 |
| **Within Groups** | 190 | 6.395 | 0.034 | - | - | 0.034 | 87 |
| **Total** | 194 | 7.323 | 0.038 | - | - | 0.039 | 100 |
| **Simple size: 50** | | | | | | | |
| **Between Groups** | 4 | 1.087 | 0.272 | 0.125 | 0.000 | 0.005 | 13 |
| **Within Groups** | 240 | 8.132 | 0.034 | - | - | 0.034 | 87 |
| **Total** | 244 | 9.219 | 0.038 | - | - | 0.039 | 100 |
| **Simple size: 60** | | | | | | | |
| **Between Groups** | 4 | 1.292 | 0.323 | 0.127 | 0.000 | 0.005 | 13 |
| **Within Groups** | 290 | 9.779 | 0.034 | - | - | 0.034 | 87 |
| **Total** | 294 | 11.071 | 0.038 | - | - | 0.039 | 100 |
