# Supplementary material for: Optimizing genomic diversity assessments for conservation of Bromus auleticus (Trinius ex Nees) using individual and pooled sequencing
Source: PLoS One. 2025 Jun 25;20(6):e0325548. doi: 10.1371/journal.pone.0325548 (PMC12194279; doi:10.1371/journal.pone.0325548)
Supplement: S2 Appendix — This document provides tables containing population genetic metrics -number of single nucleotide polymorphism (SNP) and expected heterozygosity (HE)-, two-way ANOVA, and AMOVA results across the five accessions, sample sizes, and sequencing depths. https://doi.org/10.6084/m9.figshare.28225532.v1. (DOCX) [file pone.0325548.s009.docx]

**Effects of sample size and sequencing depth on diversity and population structure analysis of *Bromus auleticus* with pool-seq dataset.**

Table A. Population genetic diversity parameters across five accessions and varying sample sizes and sequencing depths. The table presents the number of single nucleotide polymorphisms (SNPs) and expected heterozygosity (H_E_) under three sequencing depths (1.8, 3.0, and 4.8 million reads), and five sample size (20, 30, 40, 50, 60).

| **Accession** | **Sample Size** | **Sequencing depth (Mr)** | **SNPs** | **H_E_** |
| --- | --- | --- | --- | --- |
| 24 | 20 | 1.8 | 4634 | 0.373 |
| 24 | 30 | 1.8 | 5500 | 0.365 |
| 24 | 40 | 1.8 | 5913 | 0.354 |
| 24 | 50 | 1.8 | 6259 | 0.351 |
| 24 | 60 | 1.8 | 5001 | 0.368 |
| 28 | 20 | 1.8 | 6585 | 0.318 |
| 28 | 30 | 1.8 | 6641 | 0.342 |
| 28 | 40 | 1.8 | 7031 | 0.354 |
| 28 | 50 | 1.8 | 6721 | 0.346 |
| 28 | 60 | 1.8 | 8170 | 0.29 |
| 50 | 20 | 1.8 | 5259 | 0.375 |
| 50 | 30 | 1.8 | 6165 | 0.369 |
| 50 | 40 | 1.8 | 6528 | 0.37 |
| 50 | 50 | 1.8 | 6569 | 0.366 |
| 50 | 60 | 1.8 | 5453 | 0.377 |
| 87 | 20 | 1.8 | 5592 | 0.367 |
| 87 | 30 | 1.8 | 7009 | 0.343 |
| 87 | 40 | 1.8 | 6875 | 0.359 |
| 87 | 50 | 1.8 | 6999 | 0.361 |
| 87 | 60 | 1.8 | 6070 | 0.37 |
| 88 | 20 | 1.8 | 6026 | 0.354 |
| 88 | 30 | 1.8 | 6816 | 0.351 |
| 88 | 40 | 1.8 | 7142 | 0.357 |
| 88 | 50 | 1.8 | 7130 | 0.349 |
| 88 | 60 | 1.8 | 5855 | 0.359 |
| 24 | 20 | 3.0 | 766 | 0.388 |
| 24 | 30 | 3.0 | 4933 | 0.38 |
| 24 | 40 | 3.0 | 7719 | 0.374 |
| 24 | 50 | 3.0 | 10215 | 0.362 |
| 24 | 60 | 3.0 | 7422 | 0.359 |
| 28 | 20 | 3.0 | 1587 | 0.277 |
| 28 | 30 | 3.0 | 6673 | 0.344 |
| 28 | 40 | 3.0 | 8962 | 0.363 |
| 28 | 50 | 3.0 | 10515 | 0.364 |
| 28 | 60 | 3.0 | 8869 | 0.344 |
| 50 | 20 | 3.0 | 913 | 0.379 |
| 50 | 30 | 3.0 | 5423 | 0.389 |
| 50 | 40 | 3.0 | 8489 | 0.381 |
| 50 | 50 | 3.0 | 10855 | 0.351 |
| 50 | 60 | 3.0 | 8189 | 0.358 |
| 87 | 20 | 3.0 | 1022 | 0.375 |
| 87 | 30 | 3.0 | 7410 | 0.328 |
| 87 | 40 | 3.0 | 8726 | 0.36 |
| 87 | 50 | 3.0 | 10859 | 0.356 |
| 87 | 60 | 3.0 | 8183 | 0.362 |
| 88 | 20 | 3.0 | 994 | 0.379 |
| 88 | 30 | 3.0 | 5819 | 0.38 |
| 88 | 40 | 3.0 | 11004 | 0.325 |
| 88 | 50 | 3.0 | 10929 | 0.364 |
| 88 | 60 | 3.0 | 7927 | 0.368 |
| 24 | 20 | 4.8 | 1427 | 0.347 |
| 24 | 30 | 4.8 | 5151 | 0.305 |
| 24 | 40 | 4.8 | 7468 | 0.286 |
| 24 | 50 | 4.8 | 8674 | 0.28 |
| 24 | 60 | 4.8 | 6205 | 0.294 |
| 28 | 20 | 4.8 | 3627 | 0.197 |
| 28 | 30 | 4.8 | 7813 | 0.248 |
| 28 | 40 | 4.8 | 8934 | 0.282 |
| 28 | 50 | 4.8 | 9378 | 0.281 |
| 28 | 60 | 4.8 | 10025 | 0.229 |
| 50 | 20 | 4.8 | 2192 | 0.297 |
| 50 | 30 | 4.8 | 5548 | 0.327 |
| 50 | 40 | 4.8 | 7724 | 0.314 |
| 50 | 50 | 4.8 | 9276 | 0.286 |
| 50 | 60 | 4.8 | 6895 | 0.298 |
| 87 | 20 | 4.8 | 2766 | 0.26 |
| 87 | 30 | 4.8 | 8325 | 0.252 |
| 87 | 40 | 4.8 | 8577 | 0.286 |
| 87 | 50 | 4.8 | 9728 | 0.28 |
| 87 | 60 | 4.8 | 7890 | 0.286 |
| 88 | 20 | 4.8 | 2488 | 0.267 |
| 88 | 30 | 4.8 | 6437 | 0.291 |
| 88 | 40 | 4.8 | 10624 | 0.252 |
| 88 | 50 | 4.8 | 9916 | 0.274 |
| 88 | 60 | 4.8 | 7247 | 0.286 |

Table B: Two-way ANOVA results assessing the influence of sample size and sequencing depth on the number of SNPs, highlighting statistically significant outcomes. Interaction terms indicate a significant combined effect.

|  | **Df** | **Sum** | **Sq** | **Mean Sq** | **F value** | **Pr(>F)** |  |
| --- | --- | --- | --- | --- | --- | --- | --- |
| **Sample size** | 4 | 3104400 | 86 | 77610021 | 95.420 | < 2e-16 | *** |
| **Sequence depth** | 2 | 71951 | 19 | 3597559 | 4.423 | 0.0161 | * |
| **Sample size: Sequence depth** | **8** | 1148397 | 85 | 14354973 | 17.649 | 3.39E-13 | *** |
| **Residuals** | 60 | 488010 | 32 | 813351 |  |  |  |
| **Signif. codes:** | 0 ‘***’ 0.001 ‘**’ 0.01 ‘*’ 0.05 ‘.’ 0.1 ‘’ 1 | | | | | | |

Table C: Influence of sequence depth at each level of sample size in the number of SNPs with statistically significant outcomes

| **Simple size** | **Factor** | **DFn** | **DFd** | **F** | **p** | **p<0.05** | **ges** |  |
| --- | --- | --- | --- | --- | --- | --- | --- | --- |
| **20** | Sequence depth | 2 | 60 | 33.434 | 1.75E-10 | * | 0.527 |  |
| **30** | Sequence depth | 2 | 60 | 0.57 | 0.568 |  | 0.019 |  |
| **40** | Sequence depth | 2 | 60 | 9.404 | 2.8E-04 | * | 0.239 |  |
| **50** | Sequence depth | 2 | 60 | 24.819 | 1.4E-08 | * | 0.453 |  |
| **60** | Sequence depth | 2 | 60 | 6.792 | 0.002 | * | 0.185 |  |
| **Signif. codes:** | | 0 ‘***’ 0.001 ‘**’ 0.01 ‘*’ 0.05 ‘.’ 0.1 ‘’ 1 | | | | | | |

Table D: Significant difference in SNPs number by sequence depth in each sample size with statistically significant outcomes.

| **Sample size** | **Factor** | **.y.** | **group1** | **group2** | **df** | **statistic** | **p** | **p.adj** | **p.adj.signif** |  |
| --- | --- | --- | --- | --- | --- | --- | --- | --- | --- | --- |
| **20** | Sequence depth | SNPs | 1.8 | 3.0 | 60 | 7.999 | 0.000 | 0.000 | **** |  |
| **20** | Sequence depth | SNPs | 1.8 | 4.8 | 60 | 5.469 | 0.000 | 0.000 | **** |  |
| **20** | Sequence depth | SNPs | 3.0 | 4.8 | 60 | -2.531 | 0.014 | 0.042 | * |  |
| **40** | Sequence depth | SNPs | 1.8 | 3.0 | 60 | -4.001 | 0.000 | 0.001 | *** |  |
| **40** | Sequence depth | SNPs | 1.8 | 4.8 | 60 | -3.450 | 0.001 | 0.003 | ** |  |
| **50** | Sequence depth | SNPs | 1.8 | 3.0 | 60 | -6.906 | 0.000 | 0.000 | **** |  |
| **50** | Sequence depth | SNPs | 1.8 | 4.8 | 60 | -4.661 | 0.000 | 0.000 | **** |  |
| **60** | Sequence depth | SNPs | 1.8 | 3.0 | 60 | -3.521 | 0.001 | 0.002 | ** |  |
| **60** | Sequence depth | SNPs | 1.8 | 4.8 | 60 | -2.704 | 0.009 | 0.027 | * |  |
| **Signif. codes:** | 0 ‘***’ 0.001 ‘**’ 0.01 ‘*’ 0.05 ‘.’ 0.1 ‘’ 1 | | | | | | | | | |

Table E: Influence of sample size at each level of sequence depth in the number of SNPs with statistically significant outcomes.

| **Sequence depth** | **Factor** | **DFn** | **DFd** | **F** | **p** | **p<.05** | **ges** |
| --- | --- | --- | --- | --- | --- | --- | --- |
| **1.8** | Sample size | 4 | 60 | 1.325 | 0.271 |  | 0.081 |
| **3.0** | Sample size | 4 | 60 | 84.367 | 6.14E-24 | * | 0.849 |
| **4.8** | Sample size | 4 | 60 | 45.027 | 2.01E-17 | * | 0.75 |
| **Signif. codes:** | 0 ‘***’ 0.001 ‘**’ 0.01 ‘*’ 0.05 ‘.’ 0.1 ‘’ 1 | | | | | | |

Table F: Pairwise differences in SNP numbers across sample sizes for each sequencing depth. Only statistically significant outcomes are shown.

| **Sequence depth** | **Factor** | **.y.** | **group1** | **group2** | **df** | **statistic** | **p** | **p.adj** | **p.adj.signif** |
| --- | --- | --- | --- | --- | --- | --- | --- | --- | --- |
| **3.0** | Sample size | SNPs | 20 | 30 | 60 | -8.7576 | 0.0000 | 0.0000 | **** |
| **3.0** | Sample size | SNPs | 20 | 40 | 60 | -13.8916 | 0.0000 | 0.0000 | **** |
| **3.0** | Sample size | SNPs | 20 | 50 | 60 | -16.8626 | 0.0000 | 0.0000 | **** |
| **3.0** | Sample size | SNPs | 20 | 60 | 60 | -12.3804 | 0.0000 | 0.0000 | **** |
| **3.0** | Sample size | SNPs | 30 | 40 | 60 | -5.1341 | 0.0000 | 0.0000 | **** |
| **3.0** | Sample size | SNPs | 30 | 50 | 60 | -8.1050 | 0.0000 | 0.0000 | **** |
| **3.0** | Sample size | SNPs | 30 | 60 | 60 | -3.6228 | 0.0006 | 0.0060 | ** |
| **3.0** | Sample size | SNPs | 40 | 50 | 60 | -2.9710 | 0.0043 | 0.0427 | * |
| **3.0** | Sample size | SNPs | 50 | 60 | 60 | 4.4822 | 0.0000 | 0.0003 | *** |
| **4.8** | Sample size | SNPs | 20 | 30 | 60 | -7.2842 | 0.0000 | 0.0000 | **** |
| **4.8** | Sample size | SNPs | 20 | 40 | 60 | -10.8092 | 0.0000 | 0.0000 | **** |
| **4.8** | Sample size | SNPs | 20 | 50 | 60 | -12.0873 | 0.0000 | 0.0000 | **** |
| **4.8** | Sample size | SNPs | 20 | 60 | 60 | -9.0332 | 0.0000 | 0.0000 | **** |
| **4.8** | Sample size | SNPs | 30 | 40 | 60 | -3.5250 | 0.0008 | 0.0082 | ** |
| **4.8** | Sample size | SNPs | 30 | 50 | 60 | -4.8031 | 0.0000 | 0.0001 | *** |
| **4.8** | Sample size | SNPs | 50 | 60 | 60 | 3.0541 | 0.0034 | 0.0336 | * |
| **Signif. codes:** | 0 ‘***’ 0.001 ‘**’ 0.01 ‘*’ 0.05 ‘.’ 0.1 ‘’ 1 | | | | | | | | |

Table G: Influence of sequencing depth on expected heterozygosity (H_E_) based on ANOVA results.

|  | **Df** | **Sum Sq** | **Mean** | **Sq** | **F value** | **Pr(>F)** |  |
| --- | --- | --- | --- | --- | --- | --- | --- |
| **Profundidad** | 2 | 0.020218 | 0.0101 | 9 | 30.32 | 2.03E-05 | *** |
| **Residuals** | 12 | 0.004001 | 0.0003 | 33 |  |  |  |
| **Signif. codes:** | 0 ‘***’0.001 ‘**’0.01 ‘*’0.05 ‘.’ 0.1  ‘ ’1 | | | | | | |

Table H: Pairwise differences in expected heterozygosity (H_E_) between sequencing depths.

|  | **diff** | **lwr** | **upr** | **p adj** |
| --- | --- | --- | --- | --- |
| **4.8-1.8** | -0.075 | -0.11 | -0.04 | 0.00 |
| **4.8-3.0** | -0.080 | -0.11 | -0.05 | 0.00 |
| **Signif. codes:** | 0 ‘***’ 0.001 ‘**’ 0.01 ‘*’ 0.05 ‘.’ 0.1 ‘’ 1 | | | |

Table I: ANOVA results for the impact of sample size on expected heterozygosity.

|  | **Df** | **Sum Sq** | **Mean Sq** | **F value** | **Pr(>F)** |
| --- | --- | --- | --- | --- | --- |
| **Tamaño** | 4 | 0.000092 | 0.0000230 | 0.039 | 0.997 |
| **Residuals** | 20 | 0.011843 | 0.0005921 |  |  |

Table J: AMOVA results summarizing the proportion of genetic variation within and between accessions across five sample sizes and three sequencing depths.
