# Supplementary material for: Optimizing genomic diversity assessments for conservation of Bromus auleticus (Trinius ex Nees) using individual and pooled sequencing
Source: PLoS One. 2025 Jun 25;20(6):e0325548. doi: 10.1371/journal.pone.0325548 (PMC12194279; doi:10.1371/journal.pone.0325548)
Supplement: S3 Appendix — This appendix provides tables summarising ΔHE values across the five accessions, sample sizes and sequencing depths, and the associated ANOVAs. https://doi.org/10.6084/m9.figshare.28225553.v1. (DOCX) [file pone.0325548.s010.docx]

**Comparison of accession diversity between ind-seq and pool-seq datasets.**

Table A: ΔH_E_ across five accessions, sample size, and sequencing depth.

| **Accession** | **Sample size** | **Sequencing depth (Mr)** | **ΔH_E_** | **Average by sample size and sequence depth** |
| --- | --- | --- | --- | --- |
| 24 | 20 | 1.8 | 0.19 | 0.18 |
| 28 | 20 | 1.8 | 0.16 | 0.18 |
| 50 | 20 | 1.8 | 0.17 | 0.18 |
| 87 | 20 | 1.8 | 0.20 | 0.18 |
| 88 | 20 | 1.8 | 0.17 | 0.18 |
| 24 | 30 | 1.8 | 0.18 | 0.17 |
| 28 | 30 | 1.8 | 0.18 | 0.17 |
| 50 | 30 | 1.8 | 0.16 | 0.17 |
| 87 | 30 | 1.8 | 0.17 | 0.17 |
| 88 | 30 | 1.8 | 0.17 | 0.17 |
| 24 | 40 | 1.8 | 0.17 | 0.17 |
| 28 | 40 | 1.8 | 0.19 | 0.17 |
| 50 | 40 | 1.8 | 0.16 | 0.17 |
| 87 | 40 | 1.8 | 0.18 | 0.17 |
| 88 | 40 | 1.8 | 0.17 | 0.17 |
| 24 | 50 | 1.8 | 0.17 | 0.17 |
| 28 | 50 | 1.8 | 0.18 | 0.17 |
| 50 | 50 | 1.8 | 0.15 | 0.17 |
| 87 | 50 | 1.8 | 0.18 | 0.17 |
| 88 | 50 | 1.8 | 0.16 | 0.17 |
| 24 | 60 | 1.8 | 0.19 | 0.17 |
| 28 | 60 | 1.8 | 0.12 | 0.17 |
| 50 | 60 | 1.8 | 0.16 | 0.17 |
| 87 | 60 | 1.8 | 0.19 | 0.17 |
| 88 | 60 | 1.8 | 0.17 | 0.17 |
| 24 | 20 | 3.0 | 0.20 | 0.18 |
| 28 | 20 | 3.0 | 0.12 | 0.18 |
| 50 | 20 | 3.0 | 0.17 | 0.18 |
| 87 | 20 | 3.0 | 0.20 | 0.18 |
| 88 | 20 | 3.0 | 0.20 | 0.18 |
| 24 | 30 | 3.0 | 0.19 | 0.18 |
| 28 | 30 | 3.0 | 0.18 | 0.18 |
| 50 | 30 | 3.0 | 0.18 | 0.18 |
| 87 | 30 | 3.0 | 0.15 | 0.18 |
| 88 | 30 | 3.0 | 0.20 | 0.18 |
| 24 | 40 | 3.0 | 0.19 | 0.18 |
| 28 | 40 | 3.0 | 0.20 | 0.18 |
| 50 | 40 | 3.0 | 0.17 | 0.18 |
| 87 | 40 | 3.0 | 0.18 | 0.18 |
| 88 | 40 | 3.0 | 0.14 | 0.18 |
| 24 | 50 | 3.0 | 0.18 | 0.17 |
| 28 | 50 | 3.0 | 0.20 | 0.17 |
| 50 | 50 | 3.0 | 0.14 | 0.17 |
| 87 | 50 | 3.0 | 0.18 | 0.17 |
| 88 | 50 | 3.0 | 0.17 | 0.17 |
| 24 | 60 | 3.0 | 0.18 | 0.17 |
| 28 | 60 | 3.0 | 0.18 | 0.17 |
| 50 | 60 | 3.0 | 0.14 | 0.17 |
| 87 | 60 | 3.0 | 0.18 | 0.17 |
| 88 | 60 | 3.0 | 0.18 | 0.17 |
| 24 | 20 | 4.8 | 0.16 | 0.09 |
| 28 | 20 | 4.8 | 0.04 | 0.09 |
| 50 | 20 | 4.8 | 0.09 | 0.09 |
| 87 | 20 | 4.8 | 0.09 | 0.09 |
| 88 | 20 | 4.8 | 0.09 | 0.09 |
| 24 | 30 | 4.8 | 0.12 | 0.10 |
| 28 | 30 | 4.8 | 0.09 | 0.10 |
| 50 | 30 | 4.8 | 0.12 | 0.10 |
| 87 | 30 | 4.8 | 0.08 | 0.10 |
| 88 | 30 | 4.8 | 0.11 | 0.10 |
| 24 | 40 | 4.8 | 0.10 | 0.10 |
| 28 | 40 | 4.8 | 0.12 | 0.10 |
| 50 | 40 | 4.8 | 0.10 | 0.10 |
| 87 | 40 | 4.8 | 0.11 | 0.10 |
| 88 | 40 | 4.8 | 0.06 | 0.10 |
| 24 | 50 | 4.8 | 0.09 | 0.09 |
| 28 | 50 | 4.8 | 0.11 | 0.09 |
| 50 | 50 | 4.8 | 0.07 | 0.09 |
| 87 | 50 | 4.8 | 0.10 | 0.09 |
| 88 | 50 | 4.8 | 0.08 | 0.09 |
| 24 | 60 | 4.8 | 0.11 | 0.09 |
| 28 | 60 | 4.8 | 0.06 | 0.09 |
| 50 | 60 | 4.8 | 0.08 | 0.09 |
| 87 | 60 | 4.8 | 0.10 | 0.09 |
| 88 | 60 | 4.8 | 0.10 | 0.09 |

Table A: Effect of sample size on ΔH_E_ with statistically significant outcomes

|  | **Df** | **Sum Sq** | **Mean** | **Sq** | **F value** | **Pr(>F)** |  |
| --- | --- | --- | --- | --- | --- | --- | --- |
| **Profundidad** | 2 | 0.020218 | 0.0101 | 9 | 92.84 | 5E-08 | *** |
| **Residuals** | 12 | 0.001307 | 0.0001 | 9 |  |  |  |
| **Signif. codes:** | 0 ‘***’ 0.001 **’0.01 ‘*’ 0.05 ‘.’ 0.1 ‘ ’1 | | | | | | |

Table B: Influence of sequence depth on ΔH_E_

|  | **Df** | **Sum Sq** | **Mean Sq** | **F value** | **Pr(>F)** |
| --- | --- | --- | --- | --- | --- |
| **Sample size** | 4 | 0.000201 | 5.02E-05 | 0.101 | 0.981 |
| **Residuals** | 20 | 0.009960 | 4.98E-04 |  |  |
